# Steric Hindrance Drives the Boron‐Initiated Polymerization of Dienyltriphenylarsonium Ylides to Photoluminescent C5‐Polymers

**DOI:** 10.1002/anie.202109190

**Published:** 2021-09-06

**Authors:** Xin Wang, Nikos Hadjichristidis

**Affiliations:** ^1^ Physical Sciences and Engineering Division KAUST Catalysis Center Polymer Synthesis Laboratory King Abdullah University of Science and Technology (KAUST) Thuwal 23955 Saudi Arabia

**Keywords:** arsonium ylide, borane, C5 polymerization, mechanism, photoluminescence

## Abstract

A series of alkyl‐subsituted dienyltriphenylarsonium ylides were synthesized and used as monomers in borane‐initiated polymerization to obtain practically pure C5‐polymers (main‐chain grows by five carbon atoms at a time). The impact of triethylborane (Et_3_B), tributylborane (Bu_3_B), tri‐*sec*‐butylborane (*s*‐Bu_3_B), and triphenylborane (Ph_3_B) initiators on C5 polymerization was studied. Based on NMR and SEC results, we have shown that all synthesized polymers have C5 units with a unique unsaturated backbone where two conjugated double bonds are separated by one methylene. The synthesized C5‐polymers possess predictable molecular weights and narrow molecular weight distributions (*M*_n,NMR_=2.8 −11.9 kg mol^−1^, *Ð*=1.04–1.24). It has been found that increasing the steric hindrance of both the monomer and the initiator can facilitate the formation of more C5 repeating units, thus driving the polymerization to almost pure C5‐polymer (up to 95.8 %). The polymerization mechanism was studied by ^11^B NMR and confirmed by DFT calculations. The synthesized C5‐polymers are amorphous with tunable glass‐transition temperatures by adjusting the substituents of monomers, ranging from +30.1 °C to −38.4 °C. Furthermore, they possess blue photoluminescence that changes to yellow illuminating the polymers for 5 days with UV radiation of 365 nm (IIE, isomerization induced emission).

## Graphical abstract

A series of new alkyl‐substituted dienyltriphenylarsonium ylides was synthesized and used as monomers in the borane‐initiated polymerization, achieving almost pure C5‐polymer (95.8 %), with photoluminescent properties.

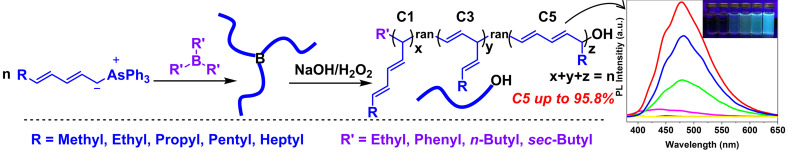

## Introduction

**Scheme 1:**
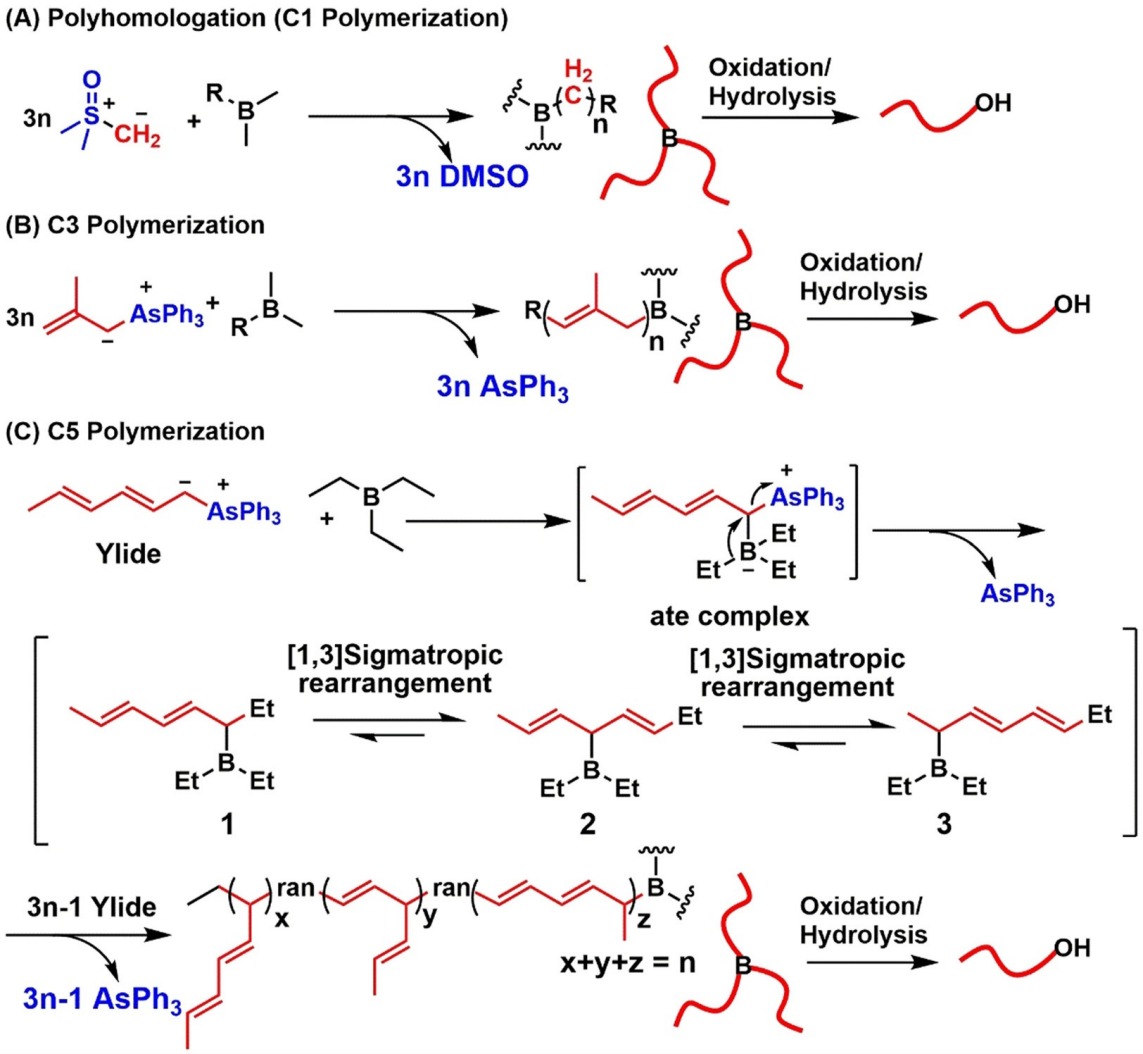
Boron‐catalyzed polymerization of ylides: A) polyhomologation (C1 polymerization); B) C3 polymerization; C) C5 polymerization.

Exploring new polymerization methods always attracts great attention because it can offer new polymeric structures with unprecedented properties. Boron‐initiated polymerization of ylides is an emerging C1 polymerization discovered by Shea et al., who coined the name polyhomologation (main chain built up by one carbon atom at a time)^[1]^ (Scheme 1 A). Polyhomologation has already been applied to the synthesis of various polymethylene‐based homo‐ and block copolymers with different topologies and properties.^[2]^ Next, a boron‐catalyzed polymerization of allylic arsonium ylides (C3 polymerization) was reported,^[3]^ where the main chain grows by three carbon atoms at a time producing C3‐polymers (e.g., polypropenylene) (Scheme 1 B). Recently, we reported a new boron‐catalyzed polymerization of ylides, which is the polymerization of dienyltriphenylarsonium ylides initiated by triethylborane (Et_3_B), called C5 polymerization (main‐chain elongation by five carbon atoms at a time) (Scheme 1 C).^[4]^

C5 polymerization has generated new polymeric materials, C5‐polymers, which have a unique unsaturated backbone structure where the two conjugated double bonds are separated by one methylene. However, in our previous work,^[4]^ the synthesized C5‐polymers contained mainly C5 (up to 84.1 %), but also C1 and C3 segments. The following possible mechanism (Scheme 1 C) has been proposed to explain the presence of C1 and C3 segments: the ylide monomer reacts with Et_3_B to form an ate complex, which by 1,2‐migration gives borane **1** with simustaneous remonval of triphenylarsine. The borane **1** may experience once or twice [1,3]‐sigmatropic rearrangement that leads to isomeric borane **2** or **3**. During the polymerization, each cycle comprises one (C3 units), two (C5 units) or none (C1 units) [1,3]‐sigmatropic rearrangement, eventually resulting in a three‐armed star terpolymer, which by oxidation/hydrolysis affords a hydroxyl‐terminated polymer. Therefore, the key to achieving a higher C5 segment ratio or even pure C5‐polymer is to ensure two [1,3]‐sigmatropic rearrangements of the intermediates in each polymerization cycle.

In this paper, a series of new alkyl‐subsituted dienyltriphenylarsonium ylides were synthesized and applied in the C5 polymerization to improve the C5 segment content. The impact of a few borane initiators, triethylborane (Et_3_B), tributylborane (Bu_3_B), tri‐*sec*‐butylborane (*s*‐Bu_3_B), and triphenylborane (Ph_3_B), at a higher C5 segment content, was studied. It was found that the steric hindrance of both the monomer and initiator promotes the two [1,3]‐sigmatropic rearrangements of the intermediates at each polymerization cycle to form more C5 segments, thus driving the polymerization to almost pure C5‐polymer. The proposed mechanism of the polymerization was studied and confirmed by nuclear magnetic resonance (NMR) and density functional theory (DFT) calculations. The thermal properties of the synthesized C5‐polymers with different substituents were also evaluated by differential scanning calorimetry (DSC) measurements. Moreover, unusual photoluminescence properties of the synthesized C5‐polymers were found by fluorescence spectroscopy.

## Results and Discussion

A series of dienyltriphenylarsonium ylide salts with different substituents on the conjugated double bond was successfully synthesized, as confirmed by ^1^H NMR, ^13^C NMR, ^19^F NMR, and ^1^H‐^1^H COSY (Scheme S1, Figures S1–S4). The corresponding ylide monomers were then generated in situ by deprotonation of the salts with *n*‐butyllithium (*n*‐BuLi) in tetrahydrofuran (THF) at −78 °C under an argon atmosphere.

**Scheme 2:**
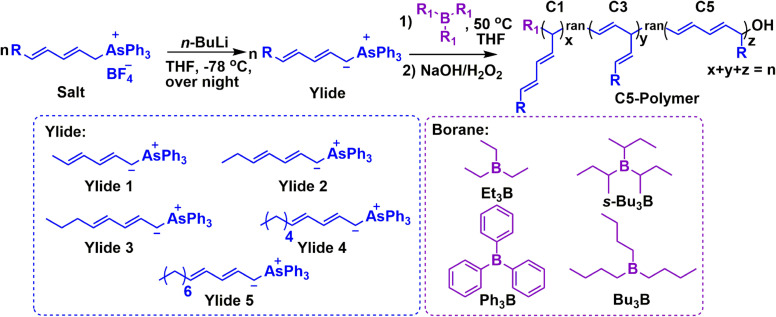
Polymerization of various dienyltriphenylarsonium ylides with different borane initiators.

Based on our previous work,^[4]^ the polymerization of ylide 1 (Scheme 2, [Ylide 1]_0_/[Et_3_B]_0_=105/1) initiated by Et_3_B at room temperature resulted in a random terpolymer (MeDEY‐1, *M*_n,NMR_=2.8 kg mol^−1^, *Ð*=1.25, Table 1, Entry 1) with a C5 segment ratio of 79.7 %. When the polymerization temperature was increased to 50 °C, the C5 segment ratio of the synthesized C5‐polymer (MeDEY‐2, *M*_n,NMR_=2.6 kg mol^−1^, *Ð*=1.14, Table 1, Entry 2; Figure S5) increases to 82.6 %, indicating that the higher temperature facilitates double [1,3]‐sigmatropic rearrangements during the polymerization. However, when the polymerization temperature was further increased to 60 °C, the amount of isolated C5‐ polymer (*M*_n,NMR_=1.7 kg mol^−1^, *Ð*=1.10, Yield: 39 %) was much lower than that of the C5‐polymer (Yield: 52 %) synthesized at 50 °C due to the decomposition of the activated ylide at 60 °C. As shown in Figure S6, the deep red Ylide 1 solution decomposed almost completely when maintained at 60 °C for 20 minutes, while there was almost no decomposition when maintained at 50 °C for 1 hour.

**Table 1:** Polymerization of various dienyltriphenylarsonium ylides initiated by different borane initiators.^[a]^

Entry	Sample	Ylide	Initiator	[Ylide]_0_/ [Initiator]_0_ (DP)	*M*_n,theory_^[b]^ [kg mol^−1^]	*M*_n,NMR_^[c]^ [kg mol^−1^]	*Ð* ^[d]^ [M_w_/M_n_]	C1^[e]^ [%]	C3^[e]^ [%]	C5^[e]^ [%]
1^[f]^	MeDEY‐1	1	Et_3_B	105/1 (35)	2.8	2.8	1.25	16.6	3.7	79.7
2	MeDEY‐2	1	Et_3_B	105/1 (35)	2.8	2.6	1.14	14.0	3.4	82.6
3	EtDEY‐1	2	Et_3_B	105/1 (35)	3.3	3.5	1.10	10.3	3.4	86.3
4	PrDEY‐1	3	Et_3_B	105/1 (35)	3.8	4.7	1.08	7.5	6.0	86.5
5	PenDEY‐1	4	Et_3_B	105/1 (35)	4.8	5.5	1.10	5.3	7.2	87.5
6	HepDEY‐1	5	Et_3_B	105/1 (35)	5.8	6.6	1.18	5.3	2.1	92.6
7	MeDEY‐3	1	Ph_3_B	105/1 (35)	2.9	2.9	1.24	11.9	3.8	84.3
8	MeDEY‐4	1	Bu_3_B	105/1 (35)	2.9	2.9	1.22	12.4	4.4	83.2
9	MeDEY‐5	1	*s‐*Bu_3_B	105/1 (35)	2.9	3.4	1.18	10.2	3.9	85.9
10	MeDEY‐6	1	*s‐*Bu_3_B	210/1 (70)	5.7	6.3	1.09	10.6	2.5	86.9
11	MeDEY‐7	1	*s‐*Bu_3_B	420/1 (140)	11.3	11.9	1.12	10.1	3.4	86.7
12	EtDEY‐2	2	*s‐*Bu_3_B	210/1 (70)	6.7	7.3	1.07	7.8	2.5	89.6
13	PrDEY‐2	3	*s‐*Bu_3_B	210/1 (70)	7.6	7.7	1.04	2.8	4.4	92.8
14	PenDEY‐2	4	*s‐*Bu_3_B	210/1 (70)	9.6	9.3	1.15	2.0	2.2	95.8
15	HepDEY‐2	5	*s‐*Bu_3_B	210/1 (70)	11.6	11.1	1.16	1.8	2.7	95.5

[a] Ylide generation conditions: −78 °C, THF, 12 hours; Polymerization conditions: 50 °C, THF. [b] Determined by the initial molar ratio of the ylide to the initiator. [c] Determined by ^1^H NMR in CDCl_3_, by comparing the integrals of the characteristic signals of methine/methylene adjacent to the hydroxyl group, at the chain end, to the saturated methine/methylene of every repeating unit of the backbone. [d] Determined by SEC in THF using PSt standards. [e] C1, C3, and C5 segment ratio of terpolymers estimated by ^1^H NMR. [f] Polymerization temperature, 25 °C.^[4]^

In order to further improve the C5 segment ratio of the C5‐polymer, the polymerizations of a series of new ylides (Ylide 2 to Ylide 5, Scheme 2) initiated by Et_3_B under the condition of [Ylide]_0_/[Et_3_B]_0_=105/1 at 50 °C (Table 1, Entries 3 to 6 ) were studied. The in situ generated ylide monomers were initially heated from −78 °C to 0 °C and stirred for 30 min, followed by the addition of Et_3_B. Then, the mixtures were placed at a 50 °C oil bath immediately to start the polymerizations. When the deep red solutions turn into colorless solutions, the ylides were entirely consumed, and the polymerizations were completed. After oxidation/hydrolysis, the corresponding hydroxyl‐terminated linear polymers were obtained. All synthesized polymers characterized by NMR and SEC (Figures S7–S10) were found to be C5‐polymers with major C5 segments. All SEC traces are symmetrical, monomodal, and narrow (*Ð*=1.08–1.18), as shown in Figures S7C‐S10C. The molecular weights of the C5‐polymers calculated by NMR were very close to the theoretical ones (Table 1, Entries 3–6). All these results indicate the living character of the Et_3_B‐initiated C5 polymerization.

Based on the ^1^H NMR and ^1^H‐^1^H COSY spectra (Figures S5 and S7‐S10), all synthesized polymers are C5‐polymers with dominant C5 segments, ranging from 82.6 % to 92.6 % (Table 1, Entries 2–6). It has been observed that when the substituents on the conjugated double bond of the ylides change from methyl, ethyl, propyl, and pentyl to heptyl, the C5 segment ratios gradually increased from 82.6 % (MeDEY‐2), 86.3 % (EtDEY‐1), 86.5 % (PrDEY‐1), and 87.5 % (PenDEY‐1) to 92.6 % (HepDEY‐1) (Table 1). This is because the long‐chain aliphatic substituent on the ylide causes a very severe steric hindrance on the side chain of the propagating polymer during the polymerization. The steric hindrance inhibits the formation of the C1 and C3 segments and promotes C5 segments. As shown in Figure S11, we obtained the optimized conformations and relative Gibbs free energy (Δ*G*) of the intermediates forming C1, C3, and C5 segments by using the dispersion‐corrected BP86 (BP86‐D3BJ) density functional theory (DFT) method with the def2tzvpp basis set and CPCM (THF) solvent model. The free energy of all C3 intermediates is higher than that of the C1 and C5 intermediates, which thermodynamically support the lowest C3 segment content in almost all synthesized polymers (Table 1). The free energy of the C1 intermediates is very close to that of the C5 intermediates. However, in all cases of Table 1, the C5 segment ratios are much higher than that of C1. This is because the formation of C5 segments reduces the crowding of the propagating three‐armed polymeric borane. Conversely, the C1 segments will produce long side chains, which will cause steric hindrance.

Furthermore, it has been observed that when the aliphatic substituents on the intermediates become longer from methyl to heptyl, the relative free energies of the C5 intermediates decrease from 1.80 kcal mol^−1^ to −0.83 kcal mol^−1^ (Figure S11a–e), which means that the C5 intermediates are more and more stable than the C1 and C3 intermediates, thus thermodynamically supporting the formation of more C5 segments. The results are consistent with the increase in the C5 segment ratios of the corresponding C5‐polymers from 82.6 % (MeDEY‐2) to 92.6 % (HepDEY‐1) (Table 1, Entries 2–6). However, the free energy difference from 1.80 kcal mol^−1^ to −0.83 kcal mol^−1^ is very small, so the energy cannot be considered as the only factor for the formation of C5 segments. In addition, the free energy of the C5 intermediate with a longer pentyl substituent is −0.52 kcal mol^−1^, which is higher than that of C5 intermediates with a propyl substituent of −0.89 kcal mol^−1^. However, the C5 segment ratio of the PenDEY‐1 (Table 1, Entry 5) is 87.5 %, which is still higher than 86.5 % of the PrDEY‐1 (Table 1, Entry 4). From all the above results, it can be concluded that the steric hindrance mainly facilitates the formation of the C5 segments, thereby driving the polymerization of dienyltriphenylarsonium ylide close to the pure C5‐polymer.

**Figure 1:**
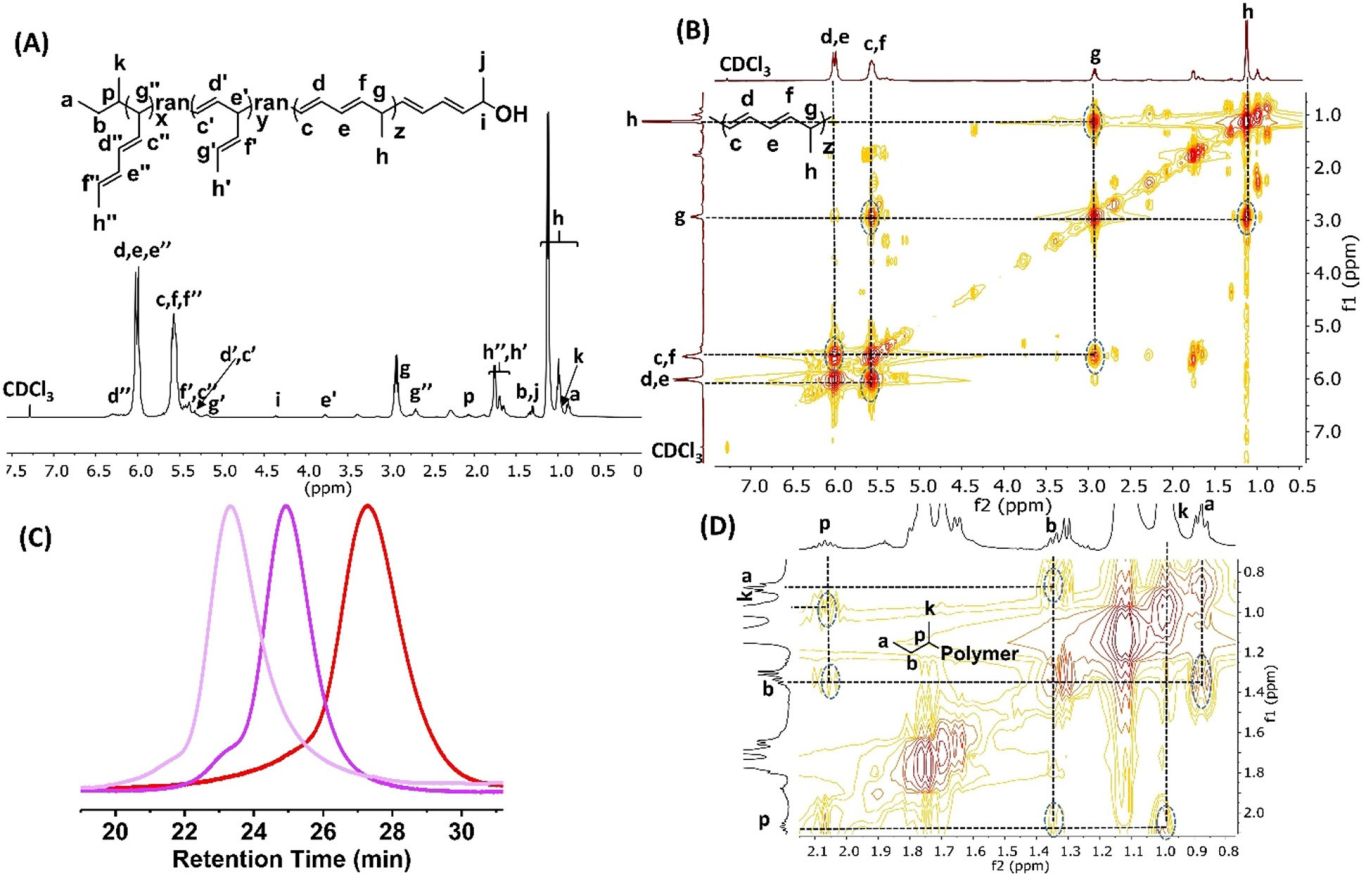
A) ^1^H NMR (CDCl_3_, 25 °C, 400 MHz), B) and D) ^1^H‐^1^H COSY (400‐400 MHz, 25 °C, CDCl_3_) spectra (Table 1, Entry 9, [Ylide 1]_0_/ [*s*‐Bu_3_B]_0_=105/1), and C) SEC traces (red line, [Ylide 1]_0_/[*s*‐Bu_3_B]_0_=105/1, Table 1, Entry 9; purple line, [Ylide 1]_0_/[*s*‐Bu_3_B]_0_=210/1, Table 1, Entry 10; pink line, [Ylide 1]_0_/[*s*‐Bu_3_B]_0_=420/1, Table 1, Entry 11) (eluent, THF; flow rate, 1.0 mL min^−1^; 25 °C) of C5‐polymer.

The influence of the borane initiator on the C5 segment ratio was also studied. In addition to Et_3_B, tributylborane (Bu_3_B), tri‐*sec*‐butylborane (*s*‐Bu_3_B), and triphenylborane (Ph_3_B) were selected as initiators for the polymerization of Ylide 1 ([Ylide 1]_0_/[borane]_0_=105/1, Table 1, Entries 7–9). All obtained polymers characterized by ^1^H NMR, ^1^H‐^1^H COSY, and SEC (Figures 1 and S12‐S13) were found to be C5‐polymers, possessing narrow molecular weight distributions (*Ð*=1.18–1.24). As shown in Table 1, the highest C5 segment ratio is 85.9 % obtained by the *s*‐Bu_3_B‐initiated polymerization of Ylide 1 (MeDEY‐5, Table 1, Entry 9), the second is 84.3 % obtained by Ph_3_B‐initiated polymerization of Ylide 1 (MeDEY‐3, Table 1, Entry 7), and the third is 83.2 % provided by Bu_3_B‐initiated polymerization of Ylide 1 (MeDEY‐4, Table 1, Entry 8). All data are higher than 82.6 % of the C5 segment ratio of the C5‐polymer synthesized by Et_3_B‐initiated polymerization of Ylide 1 (MeDEY‐2). It is because *s*‐Bu_3_B, Ph_3_B, and Bu_3_B have greater steric hindrances than Et_3_B. The larger steric hindrance will push the intermediates to undergo twice the [1,3]‐sigmatropic rearrangements forming the C5 segments for releasing the crowding at the beginning of the polymerization. Ph_3_B has the largest steric hindrance, but the strongest Lewis acidity of Ph_3_B inhibits the [1,3]‐sigmatropic rearrangement of borane, thus relatively suppressing the formation of C5 segments. In contrast, *s*‐Bu_3_B has the second‐largest steric hindrance but lower Lewis acidity, so MeDEY‐5 has the highest C5 segment ratio of 85.9 %. The relative free energy of the corresponding C5 intermediates (Figures S11a and S11f–h) calculated by DFT for the formation of the C5 segments thermodynamically supports the observed order of the C5 segment ratios: MeDEY‐5 (*s*‐Bu_3_B) > MeDEY‐3 (Ph_3_B) > MeDEY‐4 (Bu_3_B) > MeDEY‐2 (Et_3_B). The free energy of the C5 intermediate (*s*‐Bu_3_B) is the lowest (−1.27 kcal mol^−1^, Figure S11h), which is consistent with the highest C5 segments ratio (MeDEY‐5, 85.9 %). The C5 intermediate (Ph_3_B) has the second lowest free energy of −1.02 kcal mol^−1^ (Figure S11f), followed by 0.26 kcal mol^−1^ free energy (Figure S11g) of the C5 intermediate (Bu_3_B), which supports the second highest C5 segment ratio (MeDEY‐3, 84.3 %, Ph_3_B ) and the third highest C5 segment ratio (MeDEY‐4, 83.2 %, Bu_3_B). The highest free energy of the C5 intermediate (Et_3_B) is 1.80 kcal mol^−1^ (Figure S11a), which agrees with the lowest C5 segment ratio (MeDEY‐2, 82.6 %). Furthermore, the free energy of the C5 intermediates (Ph_3_B and *s*‐Bu_3_B) is even lower than that of their corresponding C1 intermediates (Figures S11f and S11h), which means that the polymerizations tend to form C5 segments rather than C1 and C3 segments in both cases. Above all, *s*‐Bu_3_B performed the best initiation in the polymerization of Ylide 1 for achieving a higher C5 segment ratio.

Figure 1 A shows a representative ^1^H NMR spectrum of the C5‐polymer (MeDEY‐5, Table 1, Entry 9) synthesized by the polymerization of Ylide 1 initiated by *s*‐Bu_3_B ([Ylide 1]_0_/[*s*‐Bu_3_B]_0_=105/1). The synthesized polymer is confirmed to be a C5‐polymer with dominant C5 segments. The characteristic signals corresponding to the C5 segments are 5.97–6.04 ppm (d, e), 5.47–5.61 ppm (c,f), 2.85–2.94 (g), and 1.09 ppm (h). The ^1^H‐^1^H COSY spectrum (Figure 1 B) further proved the structure of the C5 segments in the synthesized C5‐polymer. As shown in Figures 1 A and 1D, ^1^H NMR and ^1^H‐^1^H COSY spectra also verified the isobutyl group at the end of the polymer through end group analysis, thereby confirming the initiation by *s*‐Bu_3_B in the polymerization.

Furthermore, the polymerizations of Ylide 1 initiated by *s*‐Bu_3_B with an increased molar ratio of Ylide 1 to *s*‐Bu_3_B ([Ylide 1]_0_/[*s*‐Bu_3_B]_0_=210/1 and [Ylide 1]_0_/[*s*‐Bu_3_B]_0_=420/1, Table 1, Entries 10 and 11) were carried out. The obtained polymers are confirmed to be C5‐polymers, possessing predictable molecular weights and narrow molecular weight distributions (*M*_n,NMR_=6.3 and 11.9 kg mol^−1^, *Ð*=1.09 and 1.12). As shown in Figure 1 C, when the feeding molar ratio of Ylide 1 to *s*‐Bu_3_B increases, the SEC traces shift from the low molecular weight to high molecular weight elusion volumes, and they are all symmetrical, monomodal, and narrow. The small shoulder peaks on the SEC traces can be attributable to a small amount of double bonds on the polymer chains, especially the pendant double bonds of the C1 and C3 repeating units, which are participating in side reactions during the oxidation of the 3‐armed borane star with NaOH/H_2_O_2_.^[5]^ These results indicate the living nature of the polymerization initiated by *s*‐Bu_3_B. As summarized in Table 1, when the molecular weight of the synthesized C5‐polymer increases from 3.4 kg mol^−1^ to 6.3 kg mol^−1^, the C5 segment ratio increases from 85.9 % to 86.9 %. This is because the higher molecular weight leads to more severe crowding of the propagating polymeric borane, which will push the intermediate to undergo twice the [1,3]‐sigmatropic rearrangement to release the crowding, thus generating more C5 segments. However, when the molecular weight was further increased to 11.9 kg mol^−1^, the C5 segment ratio decreased to 86.7 %, nearly the same as the one of 6.3 kg mol^−1^. In addition, it was observed that the end of all obtained C5‐polymers is a C5 monomer unit connected to the hydroxyl group. All above results indicate that the formation of the C1 and C3 segments occurs mainly at the beginning of the polymerization, when the steric hindrance of the propagating polymeric borane is not severe at this time, thereby allowing the crowding caused by the C1 and C3 segments. When the steric hindrance caused by the molecular weight reaches a sufficient level, at the end of the polymerization, only C5 segments are produced.

The polymerization of other ylides (Ylides 2–5, Table 1, Entries 12–15, [Ylide]_0_/[*s*‐Bu_3_B]_0_=210/1) initiated by *s*‐Bu_3_B were also studied. All polymers obtained are still C5‐polymers, having predictable molecular weights and narrow polydispersity (*Ð*=1.04–1.16) as confirmed by NMR and SEC (Figures S14‐S17). Due to the higher steric hindrance of Ylides 2–5 than Ylide 1, all C5 segment ratios of the achieved C5‐polymers are higher than that of the C5‐polymer synthesized from Ylide 1, 86.9 % (MeDEY‐6, Table 1, Entry 10). The highest C5 segment ratio is 95.8 % (PenDEY‐2, Table 1, Entry 14), which comes from the polymerization of Ylide 4 initiated by *s*‐Bu_3_B. These further confirmed that the steric hindrance of the ylide monomer can increase the C5 segment ratio of the C5‐polymer. Compared to 95.8 % of PenDEY‐2, the polymerization of Ylide 5 with the higher steric hindrance produced a C5‐polymer with just 95.5 % C5 units (HepDEY‐2, Table 1, Entry 15), which means that further increasing the length of the ylide monomer alkyl side group will not result in 100 % pure C5‐polymer.

**Figure 2:**
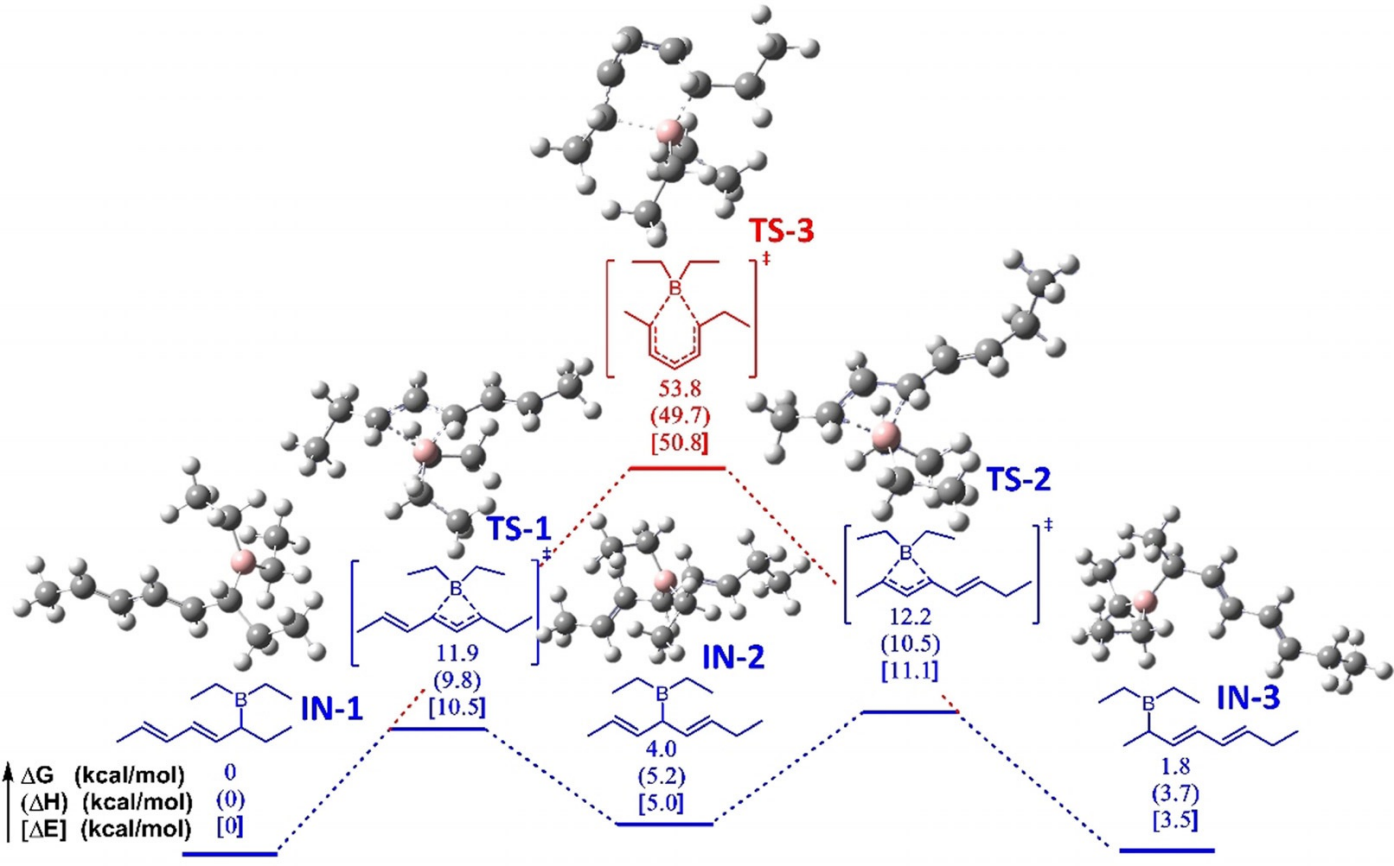
The DFT‐calculated relative Gibbs energy (Δ*G*), enthalpy (Δ*H*), and electronic energy (Δ*E*) for the rearrangement process of the Et_3_B‐initiated polymerization of Ylide 1 and optimized structures of key transition states by using the dispersion‐corrected BP86 (BP86‐D3BJ) density functional theory (DFT) method with the def2tzvpp basis set and CPCM (THF) solvent model.

In our proposed mechanism of the polymerization of dienyltriphenylarsonium ylides, the key process is the rearrangements between the three intermediates for forming the C1, C3, and C5 segments. In order to demonstrate the formation of the three intermediates, ^11^B NMR measurements of Et_3_B and a mixture of Et_3_B and Ylide 1 ([Ylide 1]_0_/[Et_3_B]_0_=1/1) at 25 °C in CDCl_3_ were carried out. As shown in Figure S18A, the characteristic boron signal of Et_3_B is a singlet at 86.94 ppm. When Et_3_B was mixed with equimolar Ylide 1, three overlapped singlets corresponding to the characteristic signals of the three produced borane intermediates are located at −1.46 ppm, −1.50 ppm, and −1.53 ppm (Figure S18B). Furthermore, the DFT calculations (Gaussian 16, BP86+D3 (BJ) DFT hybrid functional, def2tzvpp basis set, and CPCM (THF) solvent model) were performed to obtain the relative Gibbs free energy (Δ*G*), enthalpy (Δ*H*), and electronic energy (Δ*E*) for the rearrangement process of the Et_3_B‐initiated polymerization of Ylide 1 and optimized structures of key transition states, as shown in Figure 2. Based on the calculations, the C1 intermediate (IN‐1) undergoes a [1,3]‐sigmatropic rearrangement via a transition state (TS‐1) with 11.9 kcal mol^−1^ free energy barrier to form the C3 intermediate (IN‐2), which further generates the C5 intermediate (IN‐3) through a transition state (TS‐2) with 12.2 kcal mol^−1^ free energy barrier. Another reaction path in which IN‐1 may directly experience a [1,5]‐sigmatropic rearrangement to generate IN‐3 is also possible. Based on the calculation results, the IN‐1 should experience a transition state (TS‐3) with a free energy barrier of 53.8 kcal mol^−1^, which is much higher than that of the previous reaction path of 11.9 kcal mol^−1^ and 12.2 kcal mol^−1^. Therefore, the DFT calculations support that the C1 intermediate should undergo a [1,3]‐sigmatropic rearrangement leading to C3 intermediate, which will undergo a [1,3]‐sigmatropic rearrangement again to form the C5 intermediate. During the polymerization, each cycle comprises one or two [1,3]‐sigmatropic rearrangements, or none of them, which eventually leads to a three‐armed terpolymer with a small amount of C1 and C3 segments, which by oxidation/hydrolysis affords a hydroxyl‐terminated terpolymer containing the C1, C3, and C5 segments.

**Figure 3:**
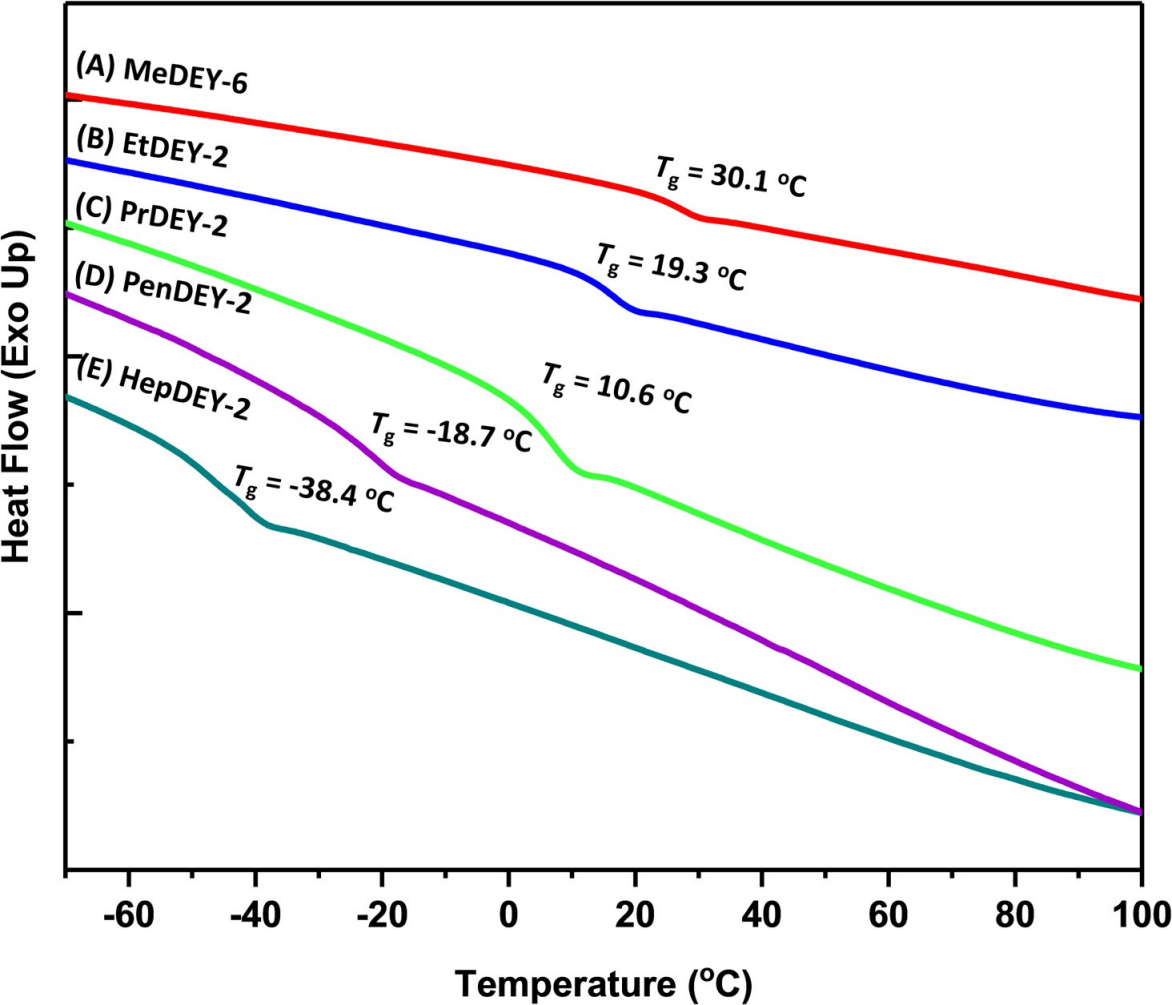
DSC traces for polymers: A) MeDEY‐6 (Table 1, Entry 10), B) EtDEY‐2 (Table 1, Entry 12), C) PrDEY‐2 (Table 1, Entry 13), D) PenDEY‐2 (Table 1, Entry 14), E) HepDEY‐2 (Table 1, Entry 15).

Differential scanning calorimetry (DSC) measurements were carried out to evaluate the thermal properties of the synthesized C5‐polymers with different substituents, including methyl (MeDEY‐6 Table 1, Entry 10), ethyl (EtDEY‐2, Table 1, Entry 12), propyl (PrDEY‐2, Table 1, Entry 13), pentyl (PenDEY‐2, Table 1, Entry 14), and heptyl (HepDEY‐2, Table 1, Entry 15), as shown in Figure 3. All C5‐polymers are amorphous, with glass‐transition temperatures (*T*_g_) ranging from −38.4 to +30.1 °C. In contrast, our group previously reported that poly(2‐methyl‐propenylene) (C3 polymerization) with an unsaturated backbone, in which the double bonds are separated by only one methylene carbon atom, has a similar structure to MeDEY‐6, but it is a crystalline polymer with a melting temperature (*T*_m_) of 47.6 °C.^[3f]^ The amorphous character of MeDEY‐6 is attributed to the irregular C1 and C3 segments randomly located on the polymer chains. From MeDEY‐6 to HepDEY‐2, the *T*_g_ gradually decrease from +30.1 °C to −38.4 °C, which is due to the enhanced plasticizing effect of longer and longer alkyl side chains. Above all, the C5‐polymers performed a tunable glass‐transition temperature by adjusting the substituents of monomers.

**Figure 4:**
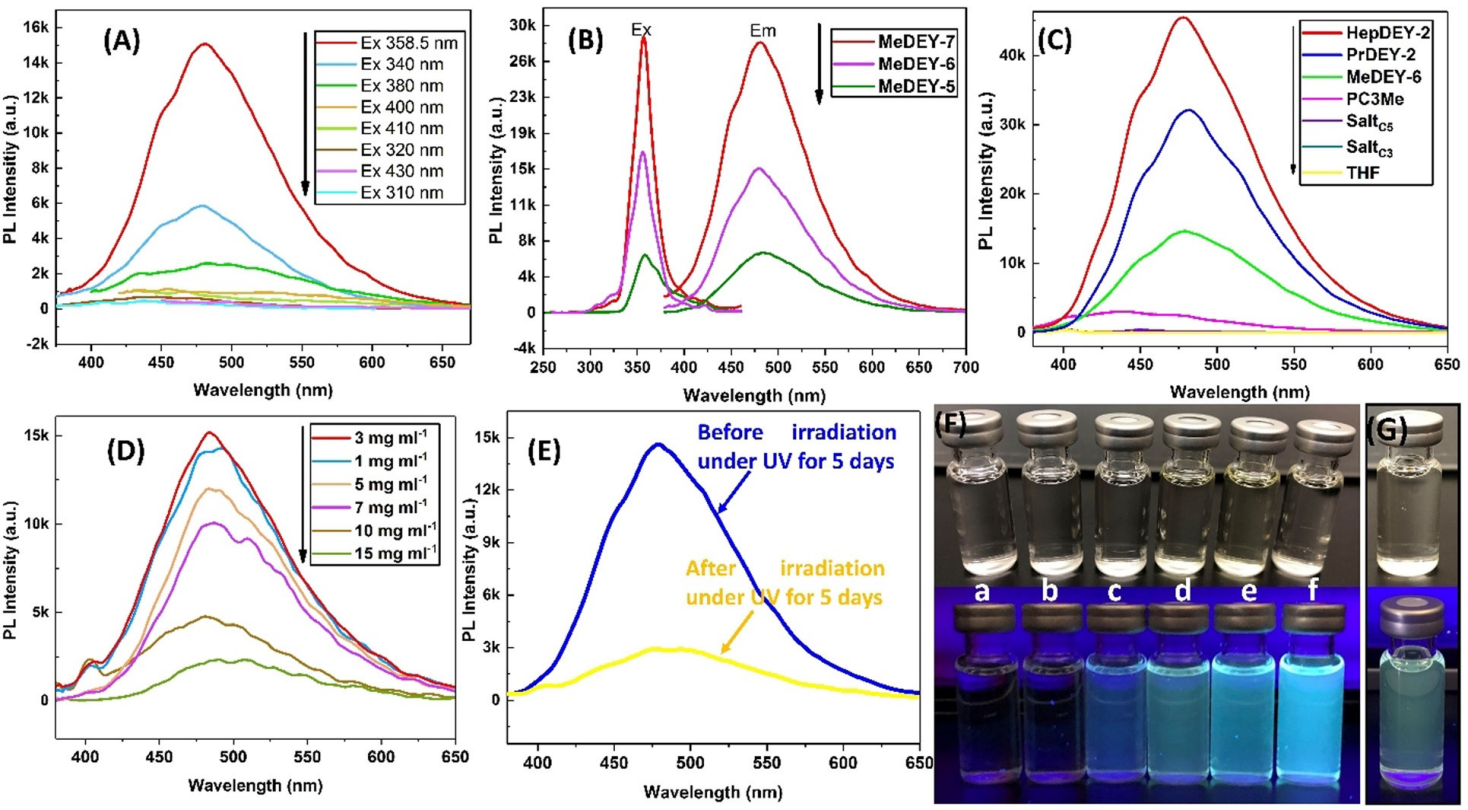
A) Fluorescence spectra of MeDEY‐7 (Table 1, Entry 11) in THF solution at varying excitation wavelengths (Concentration: 3.0 mg mL^−1^; Excitation: 310–430 nm). B) Fluorescence spectra of MeDEY‐7 (Table 1, Entry 11), MeDET‐6 (Table 1, Entry 10), and MeDEY‐5 (Table 1, Entry 9) (Concentration: 3.0 mg mL^−1^; Excitation: 358.5 nm). C) Fluorescence spectra of HepDEY‐2 (Table 1, Entry 15), PrDEY‐2 (Table 1, Entry 13), MeDEY‐6 (Table 1, Entry 10), PC3Me (poly(2‐methyl‐propenylene, *M*_n,NMR_=4.3 kg mol^−1^, *Ð*=1.07), Salt_C5_ (Salt 1, (2E,4E)‐hexa‐2,4‐dien‐1‐yl)triphenylarsonium tetrafluoroborate), Salt_C3_ (2‐methylallyl triphenylarsonium tetrafluoroborate), and THF (Solvent: THF; Concentration: 3.0 mg mL^−1^; Excitation: 358.5 nm). D) Fluorescence spectra of MeDEY‐6 in THF solution with varying concentrations (Concentration: 1.0–15.0 mg mL^−1^; Excitation: 358.5 nm). E) Fluorescence spectra of MeDEY‐6 before and after irradiation under 365 nm UV light for 5 days (Concentration: 3.0 mg mL^−1^; Excitation: 358.5 nm). F) Fluorescence images of the corresponding samples of Figure 4 C in daylight (top) and UV light (365 nm) (below) (a‐f: Salt_C3_, Salt_C5_, PC3Me, MeDEY‐6, PrDEY‐2, HepDEY‐2). G) Fluorescence images of of MeDEY‐6 before and after irradiation under 365 nm UV light for 5 days.

Previously, our group found that poly(2‐methyl‐propenylene) (double bonds separated by only one methylene) synthesized by the C3 polymerization have photoluminescence (PL) properties, which is attributed to the isomerization of the double bonds of allylic monomeric units along the polymer chain (isomerization‐induced light emission).^[3e]^ The C5‐polymers have a similar structure; the two conjugated double bonds are separated by only one methylene on the backbone, so the PL properties of the C5‐polymer were discovered as expected. Figure 4 A shows the emission spectra of C5‐polymer (MeDEY‐7, Table 1, Entry 11) in 3.0 mg mL^−1^ THF solution obtained at different excitation wavelengths (*λ*ex=310–430 nm). When *λ*ex=358.5 nm, the C5‐polymer solution exhibits the highest light emission intensity at 481.4 nm. Next, the PL properties of the C5‐polymers in 3.0 mg mL^−1^ THF solution with different molecular weights varying from MeDEY‐5 (*M*_n,NMR_=3.4 kg mol^−1^, Table 1, Entry 9), MeDEY‐6 (*M*_n,NMR_=6.3 kg mol^−1^, Table 1, Entry 10), MeDEY‐7 (*M*_n,NMR_=11.9 kg mol^−1^, Table 1, Entry 11) were examined. As shown in Figure 4 B, the emission intensity increases with increasing molecular weight. MeDEY‐7 shows the strongest PL intensity; this is because the higher molecular weight restricts intramolecular rotation, resulting in enhanced fluorescence.^[6]^

Furthermore, the PL properties of the C5‐polymers with different substituents (HepDEY‐2, Table 1, Entry 15; PrDEY‐2, Table 1, Entry 13; MeDEY‐6, Table 1, Entry 10), and poly(2‐methyl‐propenylene) (PC3Me, *M*_n,NMR_=4.3 kg mol^−1^, *Ð*=1.07) were studied. PC3Me (Figure S19) was synthesized by the C3 polymerization initiated by Et_3_B according to the literature method.^[3f]^ As shown in Figure 4 C, HepDEY‐2 has the strongest PL emission, followed by PrDEY‐2, MeDEY‐6, and PC3Me. This is because HepDEY‐2 has heptyl substituents, which is a relatively larger steric hindrance than propyl and methyl. The large steric hindrance of the side chain of the C5‐polymer suppresses the intermolecular π–π stacking interactions and thus reducing aggregation‐caused quenching (ACQ) behaviors.^[7]^ Through the fluorescence spectra of MeDEY‐6 in THF solutions of different concentrations varying from 1.0–15.0 mg mL^−1^ (Figure 4 D), ACQ behavior in C5‐polymer was also found. As shown in Figure 4 D, MeDEY‐6 solution has the strongest PL emission at 3.0 mg mL^−1^, but the lowest PL emission at 15.0 mg mL^−1^. In addition, the absence of emission from ylide precursors (triphenylarsonium salt used herein: Salt_C5_, (2E,4E)‐hexa‐2,4‐dien‐1‐yl triphenylarsonium tetrafluoroborate; Salt_C3_, 2‐methylallyl triphenylarsonium tetrafluoroborate) and THF excludes potential contaminant effect and further confirms that the emission comes from the C5‐polymers. Compared to PC3Me, the emission peaks of the C5‐polymers present a little red‐shift, as shown in Figure 4 C, which is mainly due to their original conjugated double bond chromophores.^[8]^ Figure 4 F shows the gradually increased blue fluorescence from a‐f (Salt_C3_, Salt_C5_, PC3Me, MeDEY‐6, PrDEY‐2, HepDEY‐2), consistent with the PL intensity order of Figure 4 C.

Based on our previously proposed isomerization‐induced light emission, we further conducted the irradiation of MeDEY‐6 solution (3.0 mg mL^−1^ in THF) under 365 nm UV light for 5 days. As shown in Figure 4 E, the MeDEY‐6 solution exhibits a new strong light emission at 494 nm after 5 days of UV irradiation. The red‐shift of the light emission indicates the formation of longer conjugated double bond chromophores, which is attributed to the UV‐induced isomerization. As shown in Figure S20, the MeDEY‐6 solution shows a new UV‐vis absorption band centered at around 368 nm, which is associated with π‐π* electron transition from the longer conjugated double bond system induced by isomerization. The longer conjugated double bond system may be responsible for the extensive “electron delocalization” and for a lower energy band gap, thus leading to fluorescence with a longer wavelength. Figure 4 G shows a light yellow fluorescence emitted by the MeDEY‐6 solution irradiated with 365 nm UV light for 5 days, which is consistent with the red‐shift that appears in Figure 4 E. In addition, the emission intensity of the MeDEY‐6 solution after irradiation for 5 days under 365 nm UV light is much weaker than before. This may be due to the fact that when the polymer molecule returns from the excited state to the ground state through such a lower energy band gap, more non‐radiative transitions occur.

## Conclusion

In summary, a series of new alkyl‐subsituted dienyltriphenylarsonium ylides were synthesized and used as the monomers in the borane‐initiated C5 polymerization (main‐chain grows by five carbon atoms at a time). The impact of the borane initiators, including triethylborane (Et_3_B), tributylborane (Bu_3_B), tri‐*sec*‐butylborane (*s*‐Bu_3_B), and triphenylborane (Ph_3_B) on the achievement of pure C5 polymerization, was studied. It has been found that increasing the steric hindrance of both the monomer and the initiator can facilitate the formation of more C5 repeating units, thereby driving the polymerization to almost pure C5‐polymer (the C5 repeating units up to 95.8 %). All synthesized polymers have mainly C5 repeating units (tiny C1 and C3 repeating units), possessing predictable molecular weights and narrow molecular weight distributions (*M*_n,NMR_=2.8 −11.9 kg mol^−1^, *Ð*=1.04–1.24). The key polymerization process that each cycle comprises one or two [1,3]‐sigmatropic rearrangements, or none of them, which eventually leads to the C5‐polymer having a small amount of C1 and C3 segments, was proved by NMR and DFT calculations. All synthesized C5‐polymers are amorphous with tunable glass‐transition temperatures ranging from +30.1 °C to −38.4 °C by adjusting the substituents of monomers. A photoluminescence property of C5‐polymers was discovered, which was attributed to the dual effect of their original conjugated double bond chromophores and the isomerization‐induced longer conjugated double bond chromophores. This work provides a new path to pure C5 polymerization and opens new horizons towards novel polymeric materials and properties.

## Conflict of interest

The authors declare no conflict of interest.

## Supporting information

As a service to our authors and readers, this journal provides supporting information supplied by the authors. Such materials are peer reviewed and may be re‐organized for online delivery, but are not copy‐edited or typeset. Technical support issues arising from supporting information (other than missing files) should be addressed to the authors.

Supporting Information
