# Supplementary material for: Steric Hindrance Drives the Boron‐Initiated Polymerization of Dienyltriphenylarsonium Ylides to Photoluminescent C5‐Polymers
Source: Angew Chem Int Ed Engl. 2021 Sep 6;60(41):22469–77. doi: 10.1002/anie.202109190 (PMC8518972; doi:10.1002/anie.202109190)
Supplement: Supplementary file 1 — Supporting Information [file ANIE-60-22469-s001.pdf]

Angewandte Chemie
Eine Zeitschrift der Gesellschaft Deutscher Chemiker

Supporting Information

Steric Hindrance Drives the Boron-Initiated Polymerization of Dienyltriphenylarsonium Ylides to Photoluminescent C5-Polymers

*Xin Wang and Nikos Hadjichristidis**

anie_202109190_sm_miscellaneous_information.pdf

Experimental Details

Materials

All operations of air- and moisture-sensitive chemicals and materials were carried out in flamed Schlenk-type glassware under an argon atmosphere or in an argon-filled glovebox. *n*-Butyllithium (*n*-BuLi) (1.6 M in hexane, Aldrich), triethylborane (Et_3B) (1.0 M in hexane, Aldrich), tributylborane (Bu_3B) (1.0 M in THF, Aldrich), tri-*sec*-butylborane (*s*-Bu_3B) (1.0 M in THF, Aldrich), and triphenylborane (Ph_3B) (0.25 M in THF, Aldrich) were used as received. Tetrahydrofuran was distilled over Na, then degassed three times and stored in the glovebox for use. ((2*E*,4*E*)-Hexa-2,4-dien-1-yl)triphenylarsonium tetrafluoroborate (**Salt 1**) were synthesized according to the literature method.[1] All other chemicals were purchased from Aldrich Chemicals and used as received unless otherwise stated.

Characterizations

Nuclear magnetic resonance (^{1}H NMR, ^{13}C NMR, ^{19}F NMR, and ^{1}H-^{1}H COSY) measurements were recorded on Bruker AVANCE III-400 instruments. All NMR spectra were taken in $CDCl_3$ unless otherwise stated. Size exclusion chromatography (SEC) analyses were performed using THF as an eluent at a flow rate of 1.0 mL min^{-1} on a VISCOTEK VE2001 system equipped with PSS columns (Styragel HR 2 and 4). The number-average molecular weights ($M_{n,SEC}$) and the molecular weight distribution (M_w/M_n, *Đ*) were obtained by conventional SEC analysis with a calibration curve constructed from polystyrene standards. Differential scanning calorimetry (DSC) measurements were performed using a Mettler Toledo DSC1/TC100 IntraCooler system under an inert atmosphere (nitrogen). Scans were recorded at a heating and cooling rate of 10 °C min^{-1} from -70 to +100 °C. The sample size was about 3-7 mg. Photoluminescence spectra were recorded on a Thermo Lumina Fluorescence Spectrometer.

General experimental procedures

Synthesis of dienyltriphenylarsonium ylide salts

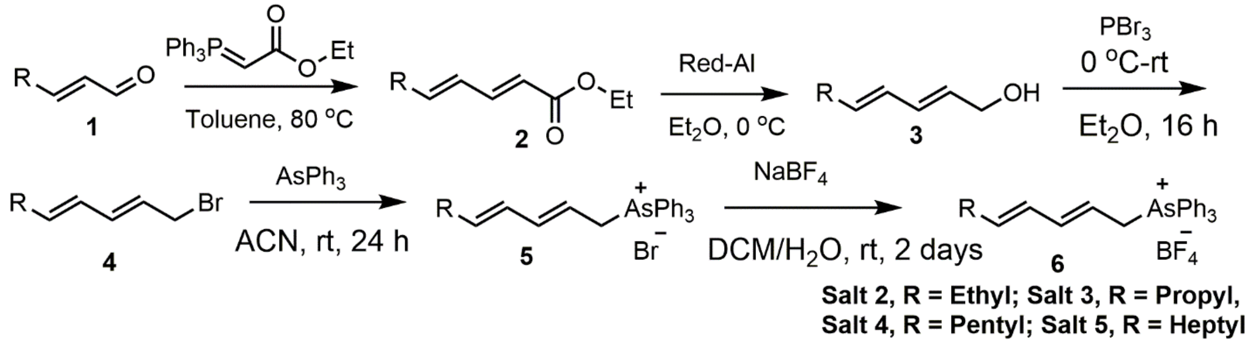

Scheme S1. Synthesis of dienyltriphenylarsonium ylide salts.

The general procedure of the synthesis of dienyltriphenylarsonium ylide salts was shown as following: (Carbethoxymethylene)triphenylphosphorane (1 eq, 48.8 mmol, 17 g) was dissolved in toluene (100 mL) followed by the addition of compound **1** (1 eq, 48.8 mmol). The mixture was heated to 80 °C for 15 hours and then cooled to room temperature. The solvent was removed under reduced pressure on a rotary evaporator. After that, hexane (30 mL) was added to precipitate Ph_3PO completely. The precipitate was filtered off and washed with hexane (3 × 30 mL). The obtained filtrate was concentrated under reduced pressure to get the crude product **2** for use in the next step without further purification.

The crude product **2** (1 eq, 42.8 mmol) was dissolved in anhydrous Et_2O (60 mL). The solution was cooled with an ice bath to 0 °C, and while intensively stirred, a solution of Red-Al in toluene (2.3 eq, 96.2 mmol, 65 wt%, 12.4 mL) was added dropwise. After 2 hours of stirring, the reaction mixture was quenched by the addition of methanol 5 ml at 0 °C. The emerged precipitate was dissolved by the addition of an aqueous solution of Potassium sodium tartrate tetrahydrate (2.3 eq, 98.4 mmol, 27.8 g/100 mL of H2O). Then the organic layer was separated, and the aqueous phase was washed with Et_2O (3 × 30 mL). The combined organic phases were dried over $MgSO_4$ and concentrated under reduced pressure to obtain the crude product **3** for use in the next step without further purification.

To a solution of the crude product **3** (100 mmol) in diethyl ether (200 ml) was added phosphorus tribromide (4.7 ml, 50 mmol) dropwise at 0 ^{o}C under argon. Then the reaction

mixture was stirred at room temperature for 16 h. The reaction mixture was cooled to 0 ^oC and quenched with ice water. The organic layer was sequentially washed by water, saturated sodium bicarbonate, and brine solution. Extracted with Et_2O, the combined organic layer was washed by brine and then dried over Na_2SO_4, filtered, and concentrated to produce the crude product **4** for use in the next step without further purification.

The crude product **4** (65 mmol) was dissolved in anhydrous ACN (90 ml), followed by the addition of $AsPh_3$ (22 g, 72 mmol). The reaction mixture was then stirred at room temperature for 24 h. The solvent was removed, and the residue was washed with Et_2O to obtain crude product **5**.

The crude product **5** (24 mmol) in 100 ml dichloromethane (DCM) and $NaBF_4$ (79 g) in 90 ml water were mixed and stirred at room temperature for two days. The aqueous layer was extracted by DCM. The combined organic layer was washed by brine and dried with Na_2SO_4, filtered, and concentrated. The crude product was purified by recrystallization (DCM/petroleum ether).

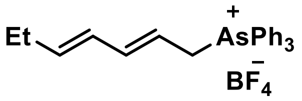

((2*E*,4*E*)-Hepta-2,4-dien-1-yl)triphenylarsonium tetrafluoroborate (Salt 2), while solid, 56% yield, 91.6% *E,E*-configuration, ^1H NMR (400 MHz, $CDCl_3$, ppm): δ 7.58-7.78 (m, 15H, h), 6.24-6.35 (m, 1H, e), 5.86-5.95 (m, 1H, d), 5.66-5.75 (m, 1H, c), 5.39-5.48 (m, 1H, f), 4.23 (d, 2H, g), 2.03 (m, 2H, b), 0.94 (t, 3H, a), see Figure S1A; ^{13}C NMR (100 MHz, $CDCl_3$, ppm): δ 140.8, 140.1, 134.2, 132.8, 130.9, 127.4, 120.9, 114.2, 29.8, 25.6, 13.1, see Figure S1B; ^{19}F NMR (377 MHz, $CDCl_3$, ppm): δ -151.94, -151.99, see Figure S1C; ^1H-^1H COSY (400-400 MHz, $CDCl_3$, ppm) see Figure S1D.

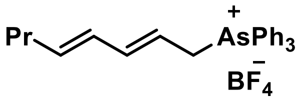

((2*E*,4*E*)-Octa-2,4-dien-1-yl)triphenylarsonium tetrafluoroborate (Salt 3), white solid, 43% yield, 90.0% *E,E*-configuration, ^1H NMR (400 MHz, $CDCl_3$, ppm): δ 7.58-7.79 (m, 15H, i), 6.24-6.33 (m, 1H, f), 5.85-5.96 (m, 1H, e), 5.59-5.69 (m, 1H, d), 5.37-5.48 (m, 1H, g), 4.23 (d, 2H, h), 1.98 (m, 2H, c), 1.34 (m, 2H, b), 0.84 (t, 3H, a), see Figure S2A; ^{13}C NMR (100 MHz, $CDCl_3$, ppm): δ 140.8, 138.5, 134.2, 132.8, 130.9, 128.5, 120.9, 114.2, 34.6, 29.8, 22.1, 13.7, see Figure S2B; ^{19}F NMR (377 MHz, $CDCl_3$, ppm): δ -151.97, -152.02, see Figure S2C; ^1H-^1H COSY (400-400 MHz, $CDCl_3$, ppm) see Figure S2D.

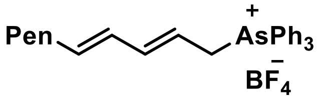

((2*E*,4*E*)-Deca-2,4-dien-1-yl)triphenylarsonium tetrafluoroborate (Salt 4), white solid, 64% yield, 94% *E,E*-configuration, ^{1}H NMR (400 MHz, $CDCl_3$, ppm): δ 7.54-7.80 (m, 15H, k), 6.21-6.36 (m, 1H, h), 5.85-5.94 (m, 1H, g), 5.60-5.70 (m, 1H, f), 5.35-5.48 (m, 1H, i), 4.22 (d, 2H, j), 1.99 (m, 2H, e), 1.16-1.36 (m, 6H, b,c,d), 0.86 (t, 3H, a), see Figure S3A; ^{13}C NMR (100 MHz, $CDCl_3$, ppm): δ 140.8, 138.8, 134.2, 132.8, 130.9, 128.3, 120.9, 114.2, 32.6, 31.3, 29.8, 28.5, 22.4, 14.0, see Figure S3B; ^{19}F NMR (377 MHz, $CDCl_3$, ppm): δ -151.95, -152.01, see Figure S3C; ^{1}H-^{1}H COSY (400-400 MHz, $CDCl_3$, ppm) see Figure S3D.

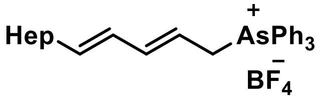

((2*E*,4*E*)-Dodeca-2,4-dien-1-yl)triphenylarsonium tetrafluoroborate (Salt 5), white solid, 59%, 92% *E,E*-configuration, ^{1}H NMR (400 MHz, $CDCl_3$, ppm): δ 7.58-7.78 (m, 15H, n), 6.24-6.34 (m, 1H, j), 5.82-5.94 (m, 1H, i), 5.61-5.71 (m, 1H, h), 5.38-5.48 (m, 1H, k), 4.25 (d, 2H, m), 2.00 (m, 2H, g), 1.16-1.37 (m, 10H, b,c,d,e,f), 0.87 (t, 3H, a), see Figure S4A; ^{13}C NMR (100 MHz, $CDCl_3$, ppm): δ 140.88, 138.84, 134.18, 132.84, 130.88, 128.33, 120.95, 114.14, 32.63, 31.79, 29.81, 29.11, 29.09, 28.90, 22.63, 14.11, see Figure S4B; ^{19}F NMR (377 MHz, $CDCl_3$, ppm): δ -151.97, -152.02, see Figure S4C; ^{1}H-^{1}H COSY (400-400 MHz, $CDCl_3$, ppm) see Figure S4D.

General polymerization of dienyltriphenylarsonium ylides

A typical C5 polymerization procedure (**Ylide 1**, Table 1, Entry 2) is given below: A suspension of dienyltriphenylarsonium ylide **salt 1** (1.52 g, 3.2 mmol) in THF (30 ml) was cooled to -78 °C. Then, *n*-BuLi (2 ml, 1.6 M in hexane) was added dropwise to the suspension. Stirring was continued overnight and then 30 minutes at 0 °C. To the obtained red solution, triethylborane (30 µL, 1.0 M in hexane) was added quickly, and then the mixture was placed at 50 °C immediately. The reaction occurred with an instantaneous discoloration of the solution. After discoloration, the solution was placed with H_2O_2/NaOH for oxidation/hydrolysis. The solvent was then removed under a vacuum. The residue was dissolved in dichloromethane. The polymer was obtained by precipitation in methanol (containing 10 mg / L butylated hydroxytoluene as the antioxidant) three times, where $AsPh_3$ is soluble and therefore easily removed from the polymer. The polymer was dried by the vacuum oven at room temperature for 24 h and then stored in a refrigerator at -20 °C.

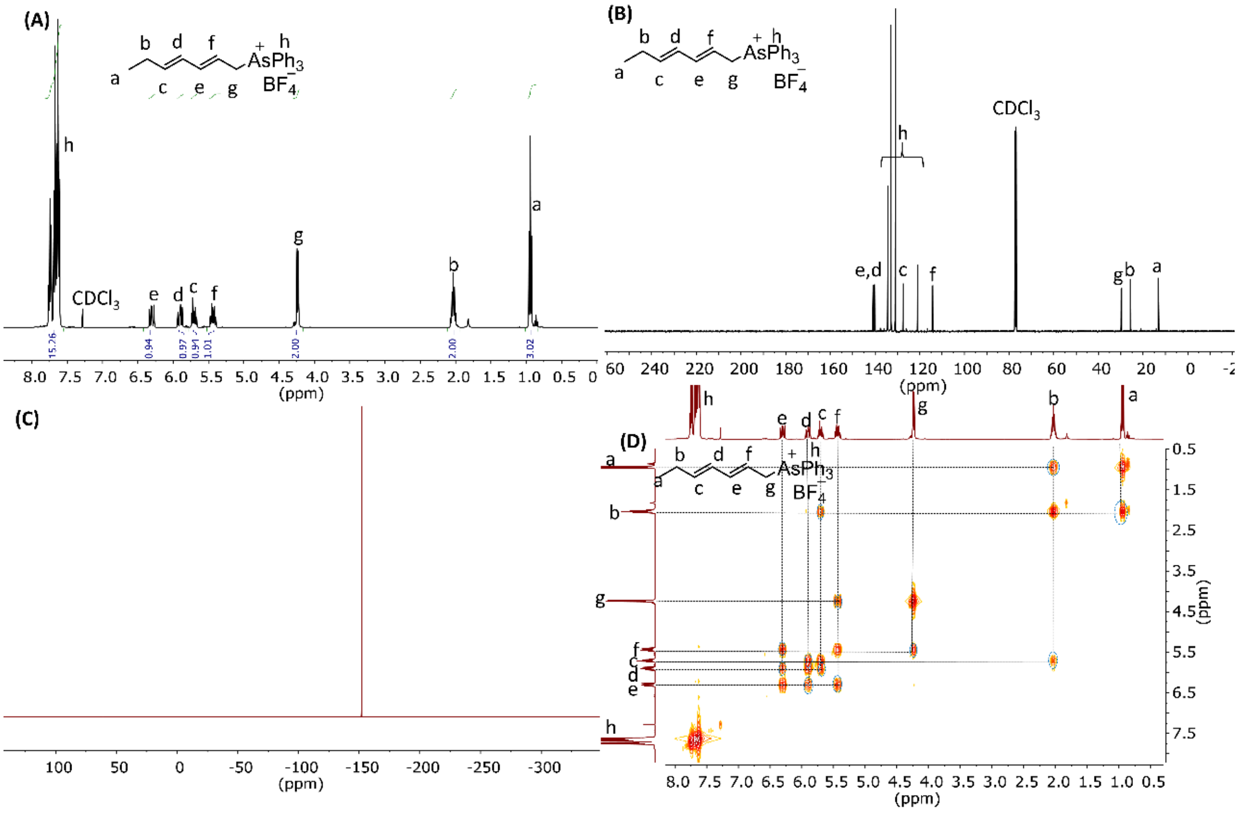

Figure S1. (A) ^{1}H NMR ($CDCl_3$, 25 ^{o}C, 400 MHz), (B) ^{13}C NMR ($CDCl_3$, 25 ^{o}C, 100 MHz), (C) ^{19}F NMR (377 MHz, 25 ^{o}C, $CDCl_3$), and (D) ^{1}H-^{1}H COSY (400-400 MHz, 25 ^{o}C, $CDCl_3$) spectra of ((2*E*,4*E*)-hepta-2,4-dien-1-yl)triphenylarsonium tetrafluoroborate (Salt 2).

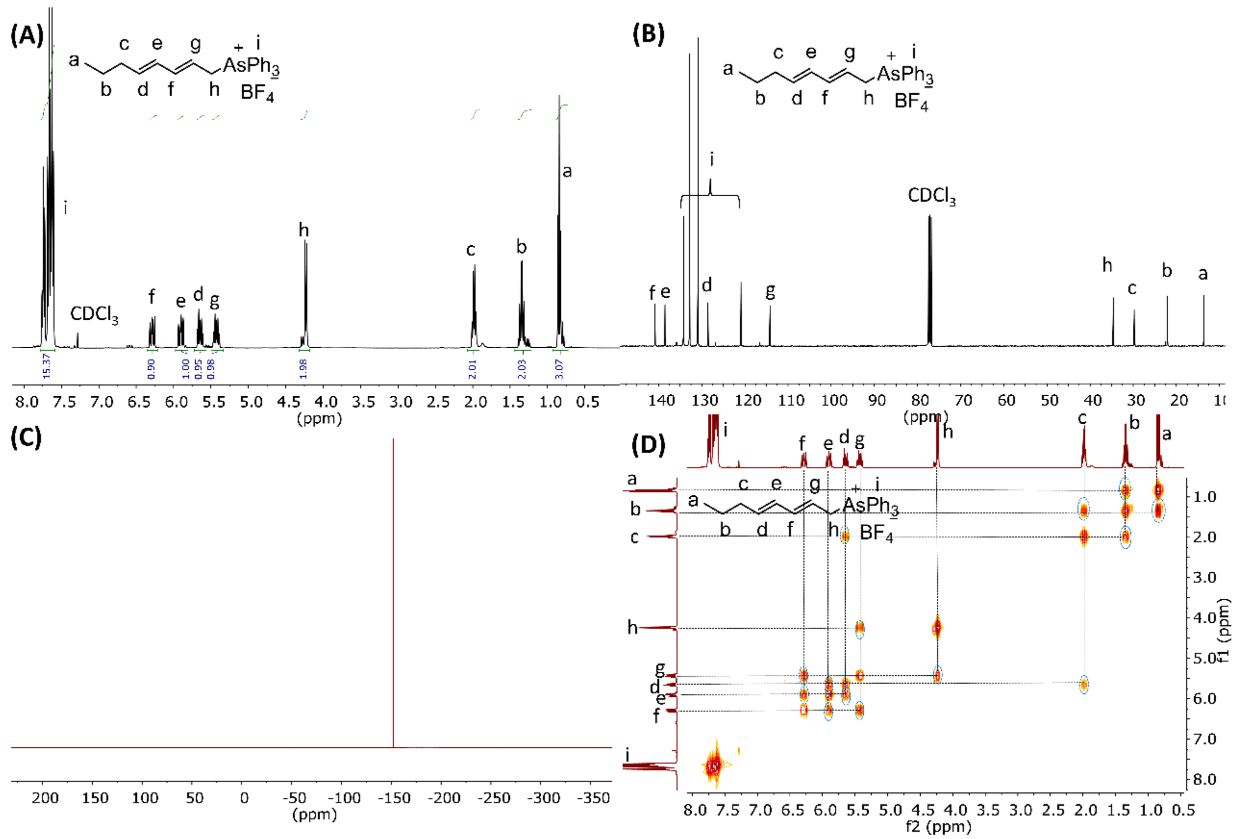

Figure S2. (A) ^{1}H NMR (CDCl$_3$, 25 °C, 400 MHz), (B) ^{13}C NMR (CDCl$_3$, 25 °C, 100 MHz), (C) ^{19}F NMR (377 MHz, 25 °C, CDCl$_3$), and (D) ^{1}H-^{1}H COSY (400-400 MHz, 25 °C, CDCl$_3$) spectra of ((2*E*,4*E*)-octa-2,4-dien-1-yl)triphenylarsonium tetrafluoroborate (Salt 3).

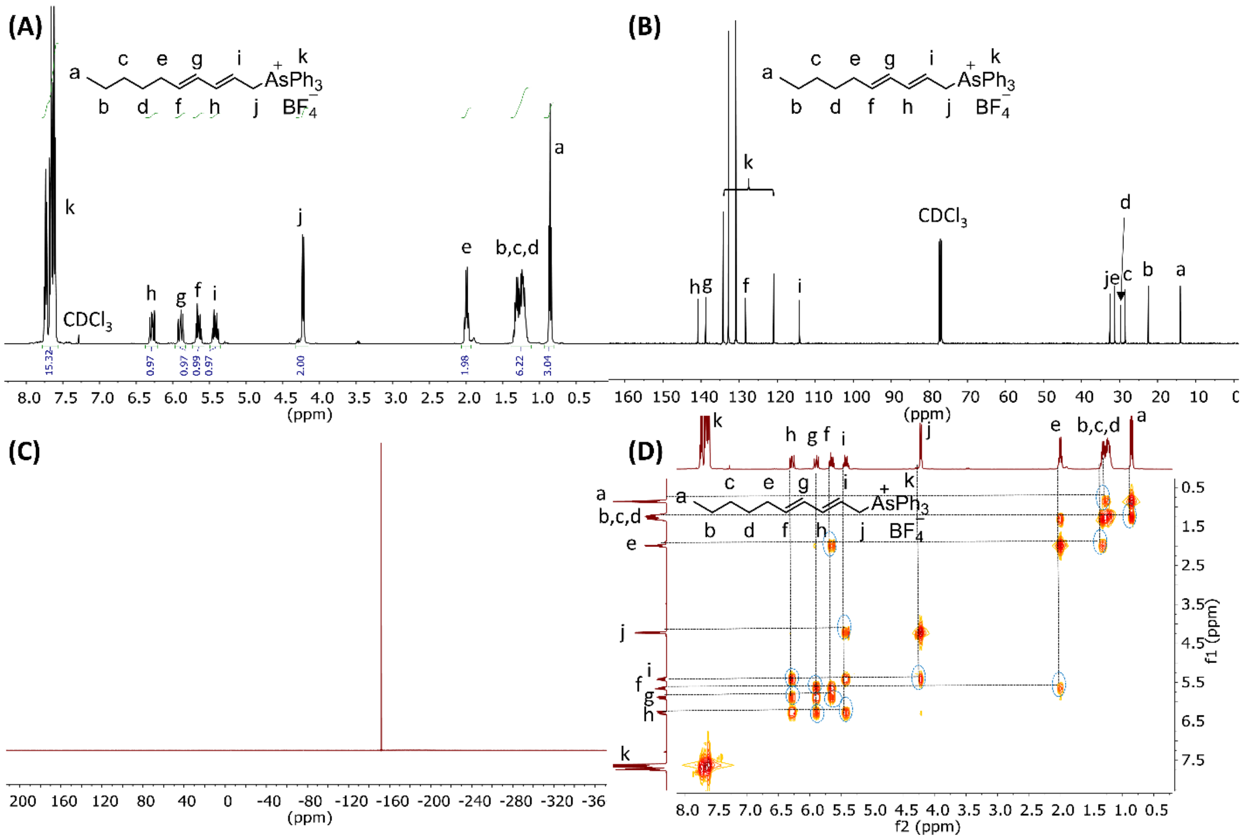

Figure S3. (A) ^{1}H NMR ($CDCl_3$, 25 °C, 400 MHz), (B) ^{13}C NMR ($CDCl_3$, 25 °C, 100 MHz), (C) ^{19}F NMR (377 MHz, 25 °C, $CDCl_3$), and (D) ^{1}H-^{1}H COSY (400-400 MHz, 25 °C, $CDCl_3$) spectra of ((2*E*,4*E*)-deca-2,4-dien-1-yl)triphenylarsonium tetrafluoroborate (Salt 4).

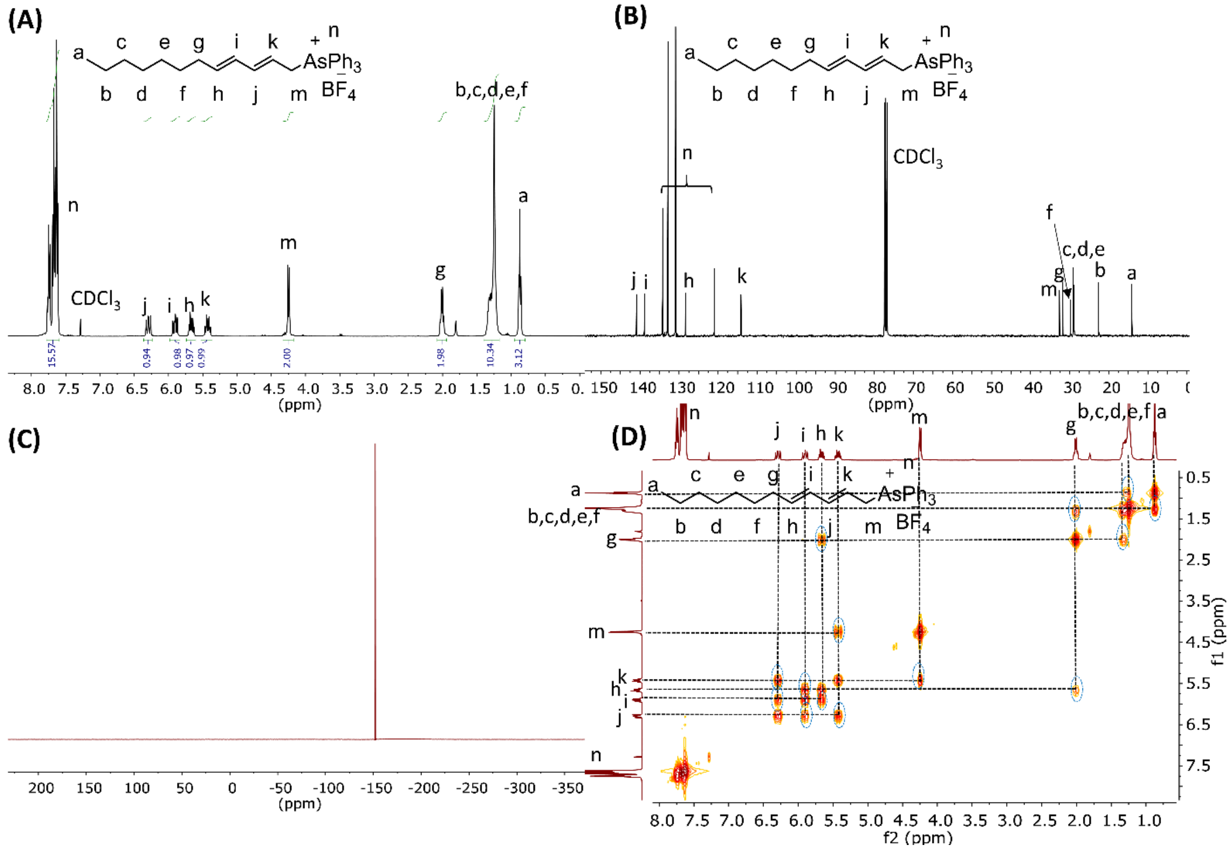

Figure S4. (A) ^{1}H NMR ($CDCl_3$, 25 °C, 400 MHz), (B) ^{13}C NMR ($CDCl_3$, 25 °C, 100 MHz), (C) ^{19}F NMR (377 MHz, 25 °C, $CDCl_3$), and (D) ^{1}H-^{1}H COSY (400-400 MHz, 25 °C, $CDCl_3$) spectra of ((2*E*,4*E*)-dodeca-2,4-dien-1-yl)triphenylarsonium tetrafluoroborate (Salt 5).

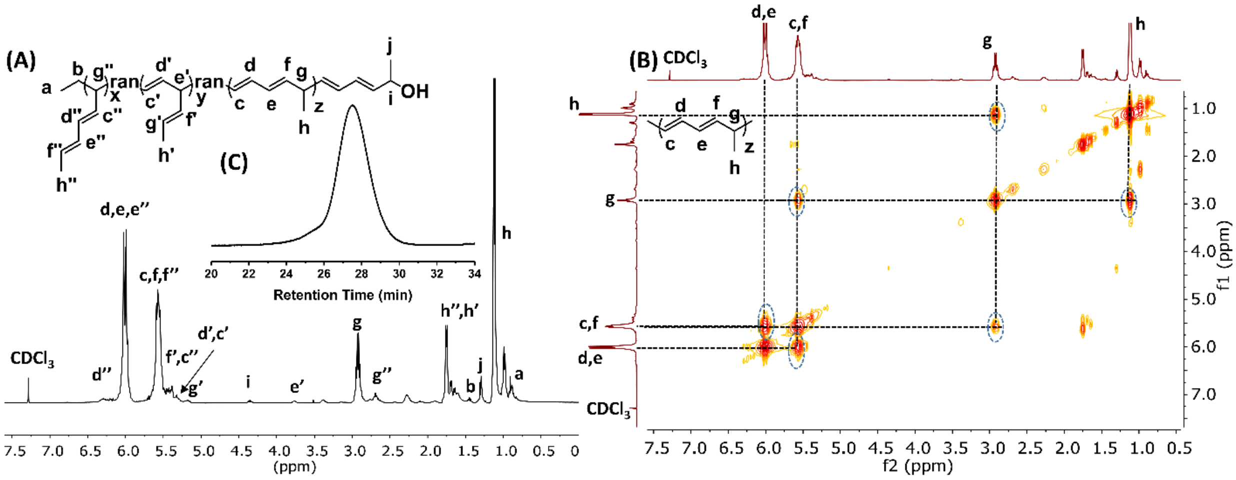

Figure S5. (A) ^{1}H NMR ($CDCl_3$, 25 ^{o}C, 400 MHz), (B) ^{1}H-^{1}H COSY (400-400 MHz, 25 ^{o}C, $CDCl_3$) spectra and (C) SEC (eluent, THF; flow rate, 1.0 ml min^{-1}; 25 ^{o}C) trace of C5-polymer (Table 1, Entry 2, [Ylide 1]$_0$/[Et_3B]$_0$ = 105/1).

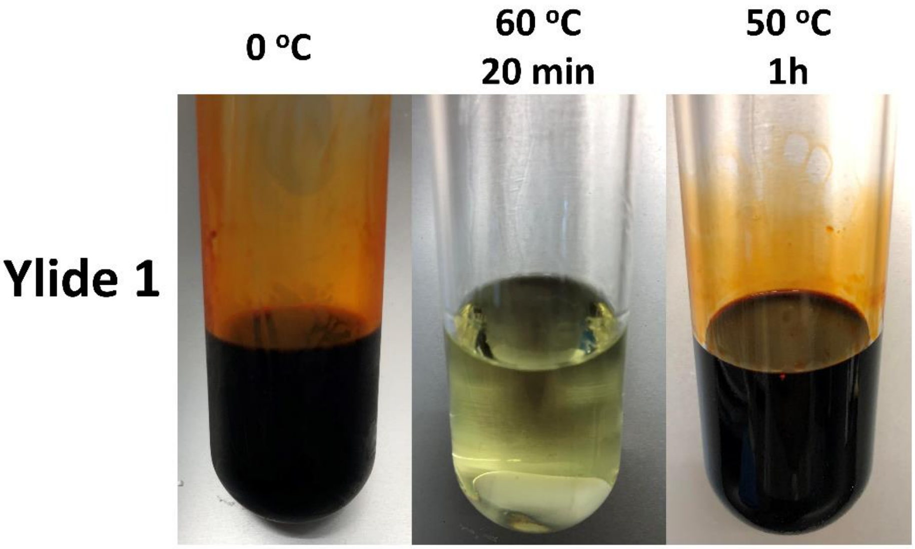

Figure S6. Pictures of the ylide 1 solution at 0 ^{o}C, at 60 ^{o}C maintained for 20 minutes, and at 50 ^{o}C for 1h.

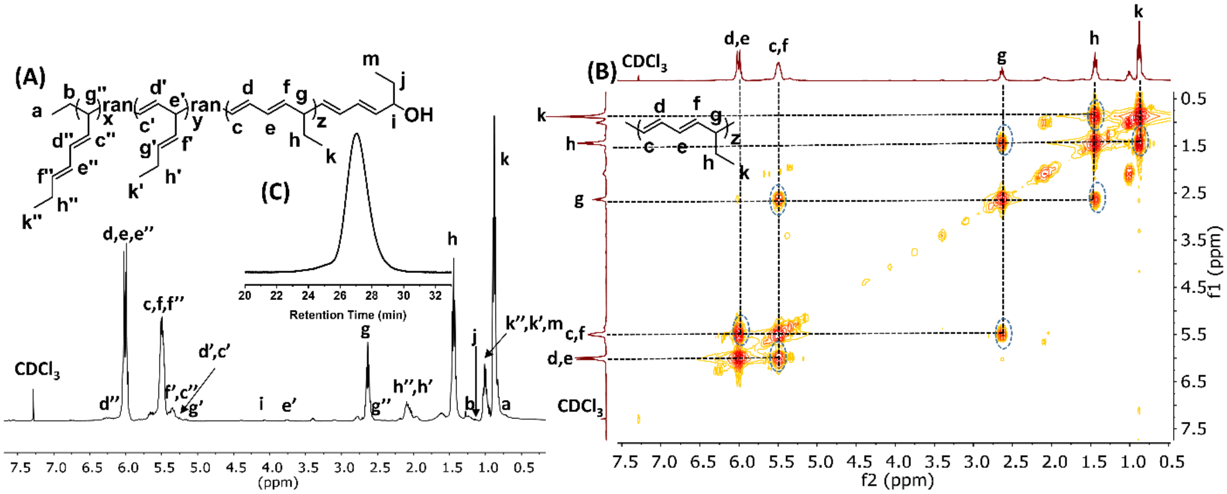

Figure S7. (A) ^{1}H NMR ($CDCl_3$, 25 ^{o}C, 400 MHz), (B) ^{1}H-^{1}H COSY (400-400 MHz, 25 ^{o}C, $CDCl_3$) spectra and (C) SEC (eluent, THF; flow rate, 1.0 ml min^{-1}; 25 ^{o}C) trace of C5-polymer (Table 1, Entry 3, [Ylide 2]$_0$/[Et_3B]$_0$ = 105/1).

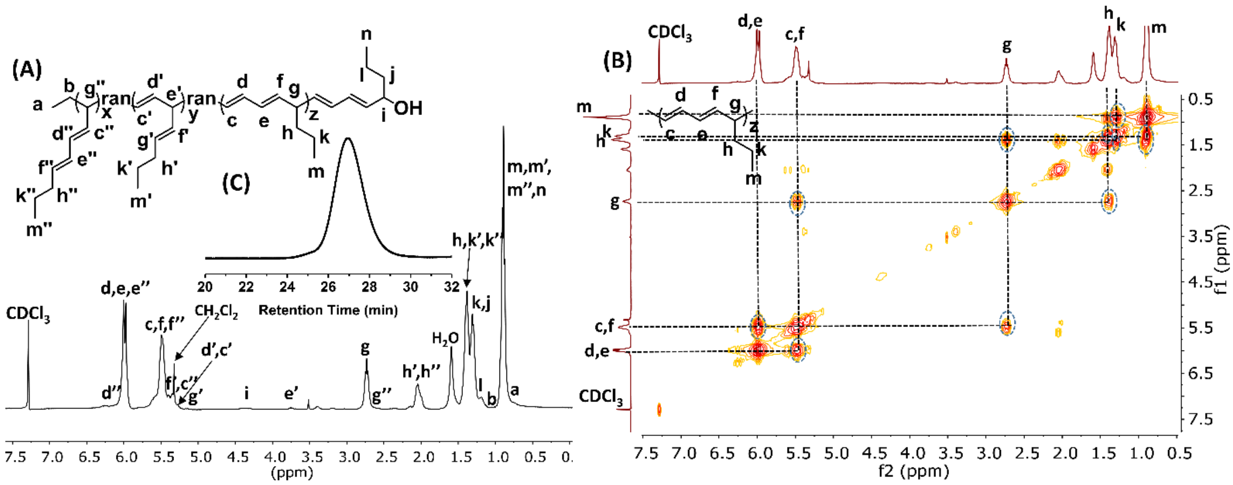

Figure S8. (A) ^{1}H NMR ($CDCl_3$, 25 °C, 400 MHz), (B) ^{1}H-^{1}H COSY (400-400 MHz, 25 °C, $CDCl_3$) spectra and (C) SEC (eluent, THF; flow rate, 1.0 ml min^{-1}; 25 °C) trace of C5-polymer (Table 1, Entry 4, $[\text{Ylide 3}]_0/[Et_3B]_0$ = 105/1).

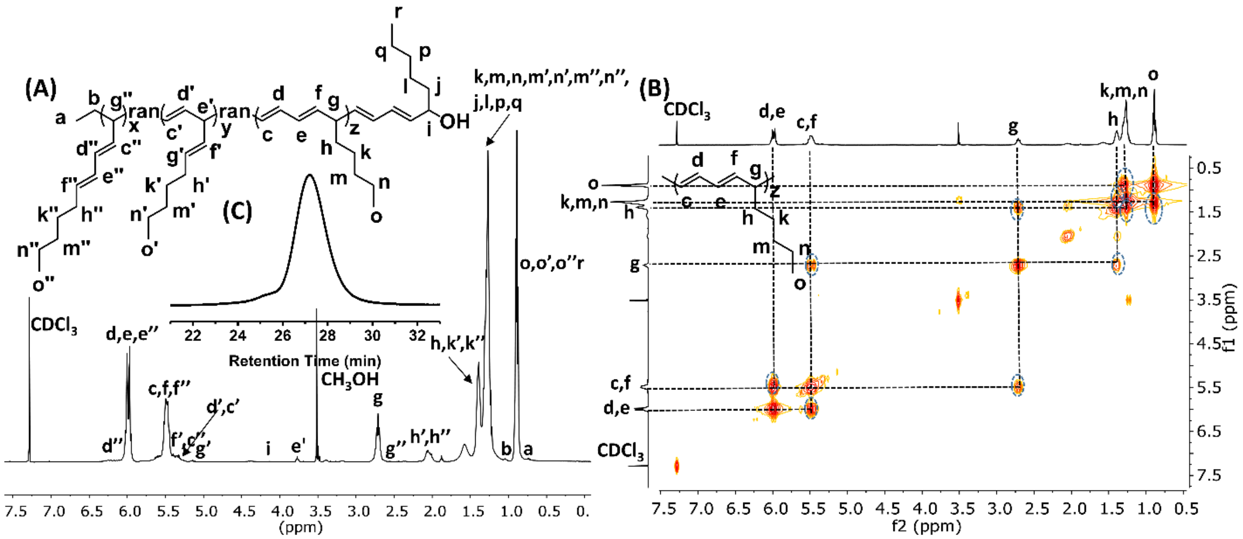

Figure S9. (A) ^{1}H NMR ($CDCl_3$, 25 °C, 400 MHz), (B) ^{1}H-^{1}H COSY (400-400 MHz, 25 °C, $CDCl_3$) spectra and (C) SEC (eluent, THF; flow rate, 1.0 ml min^{-1}; 25 °C) trace of C5-polymer (Table 1, Entry 5, $[\text{Ylide 4}]_0/[Et_3B]_0$ = 105/1).

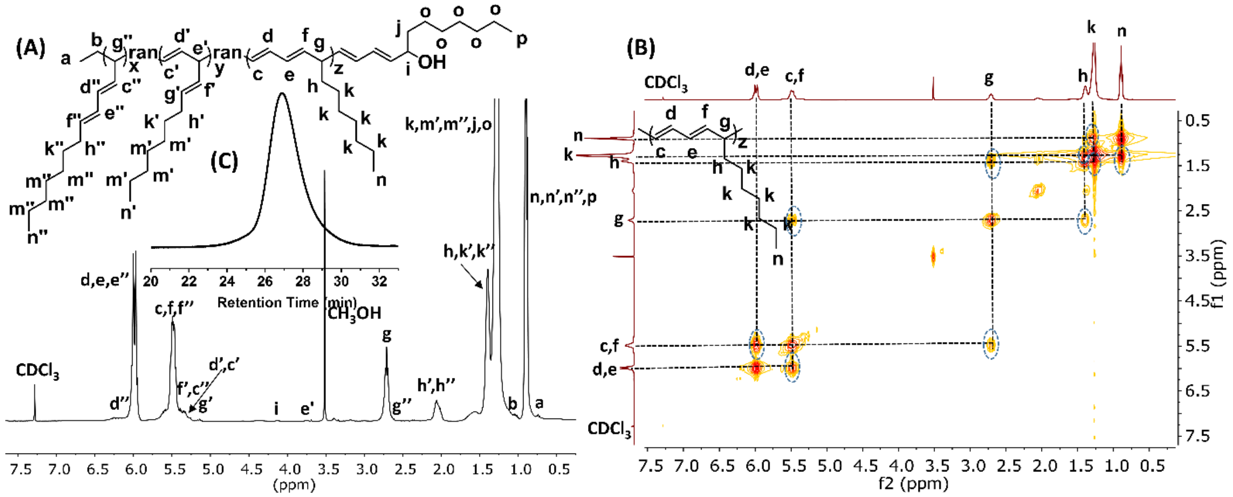

Figure S10. (A) ^{1}H NMR ($CDCl_3$, 25 °C, 400 MHz), (B) ^{1}H-^{1}H COSY (400-400 MHz, 25 °C, $CDCl_3$) spectra and (C) SEC (eluent, THF; flow rate, 1.0 ml min^{-1}; 25 °C) trace of C5-polymer (Table 1, Entry 6, [Ylide 5]$_0$/[Et_3B]$_0$ = 105/1).

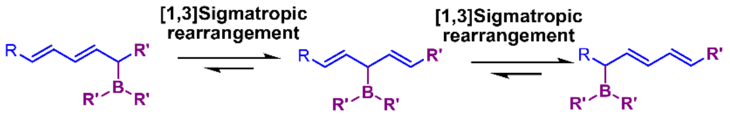

Computed relative Gibbs free energy	C1 Intermediate ΔG (kcal mol^{-1})	C3 Intermediate ΔG (kcal mol^{-1})	C5 Intermediate ΔG (kcal mol^{-1})
a. R = Methyl; R' = Ethyl	0	3.97	1.80
b. R = Ethyl; R' = Ethyl	0	3.44	-0.60
c. R = Propyl; R' = Ethyl	0	3.15	-0.89
d. R = Pentyl; R' = Ethyl	0	3.64	-0.52
e. R = Heptyl; R' = Ethyl	0	3.42	-0.83
f. R = Methyl; R' = Phenyl	0	3.26	-1.02
g. R = Methyl; R' = *n*-Butyl	0	4.87	0.26
h. R = Methyl; R' = *sec*-Butyl	0	1.61	-1.27

Figure S11. Computed relative Gibbs free energy (ΔG) of the intermediates generated in borane initiated the polymerization of ylide by using the dispersion-corrected BP86 (BP86-D3BJ) density functional theory (DFT) method with the def2tzvpp basis set and CPCM (THF) solvent model.

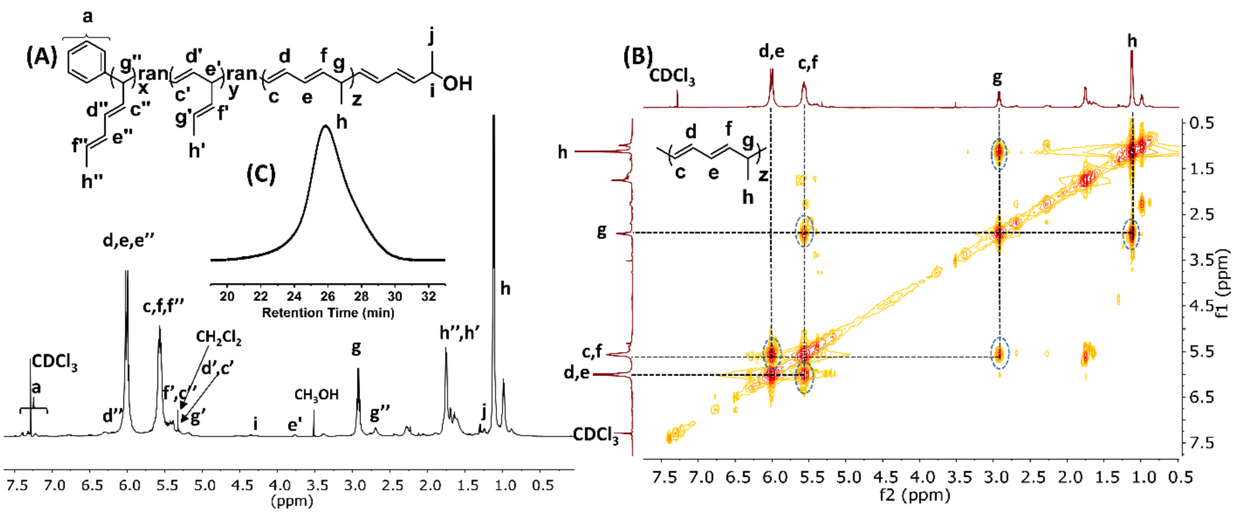

Figure S12. (A) ^{1}H NMR ($CDCl_3$, 25 °C, 400 MHz), (B) ^{1}H-^{1}H COSY (400-400 MHz, 25 °C, $CDCl_3$) spectra and (C) SEC (eluent, THF; flow rate, 1.0 ml min^{-1}; 25 °C) trace of C5-polymer (Table 1, Entry 7, $[Ylide\ 1]_0/[Ph_3B]_0$ = 105/1).

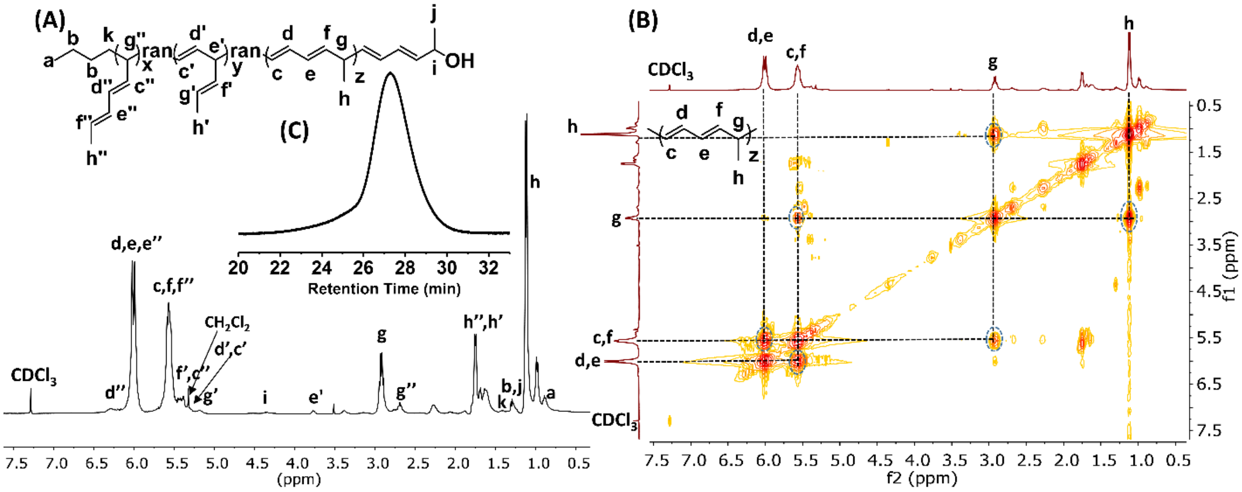

Figure S13. (A) ^{1}H NMR ($CDCl_3$, 25 °C, 400 MHz), (B) ^{1}H-^{1}H COSY (400-400 MHz, 25 °C, $CDCl_3$) spectra and (C) SEC (eluent, THF; flow rate, 1.0 ml min^{-1}; 25 °C) trace of C5-polymer (Table 1, Entry 8, $[Ylide\ 1]_0/[Bu_3B]_0$ = 105/1).

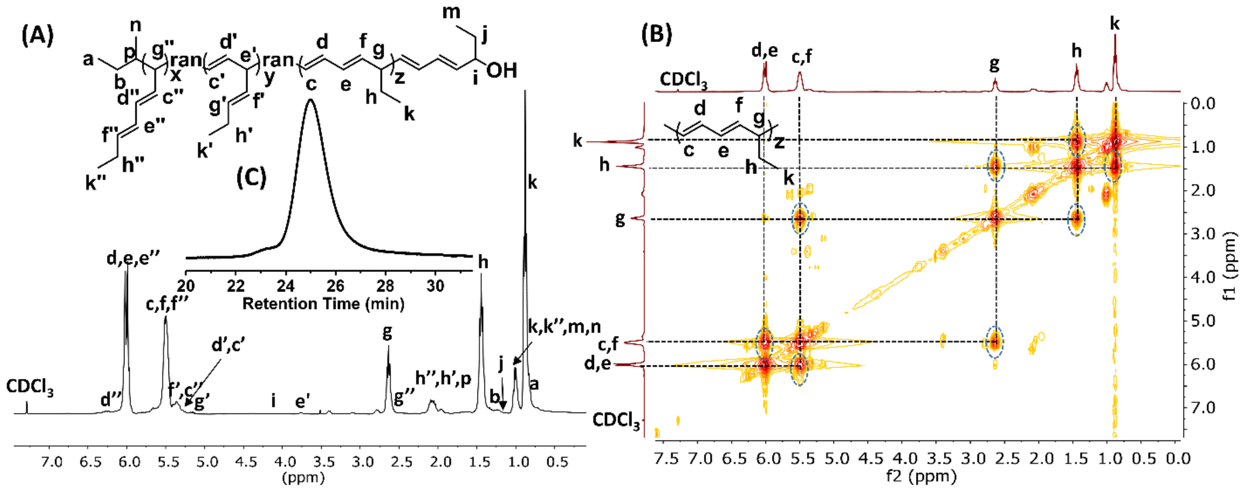

Figure S14. (A) ^{1}H NMR ($CDCl_3$, 25 °C, 400 MHz), (B) ^{1}H-^{1}H COSY (400-400 MHz, 25 °C, $CDCl_3$) spectra and (C) SEC (eluent, THF; flow rate, 1.0 ml min^{-1}; 25 °C) trace of C5-polymer (Table 1, Entry 12, [Ylide 2]$_0$/[*s*-Bu_3B]$_0$ = 210/1).

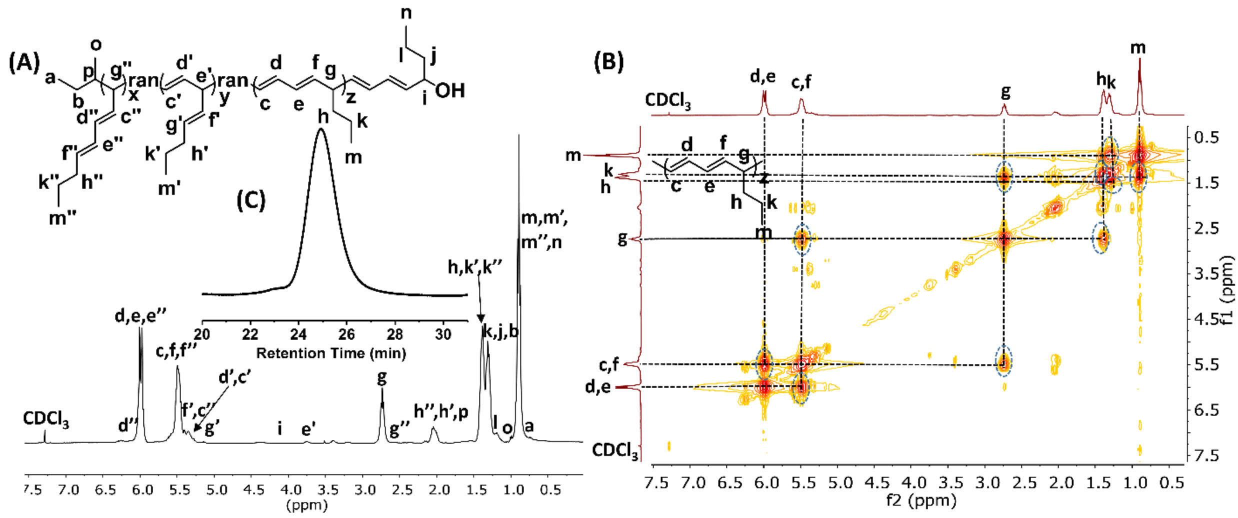

Figure S15. (A) ^{1}H NMR ($CDCl_3$, 25 °C, 400 MHz), (B) ^{1}H-^{1}H COSY (400-400 MHz, 25 °C, $CDCl_3$) spectra and (C) SEC (eluent, THF; flow rate, 1.0 ml min^{-1}; 25 °C) trace of C5-polymer (Table 1, Entry 13, [Ylide 3]$_0$/[*s*-Bu_3B]$_0$ = 210/1).

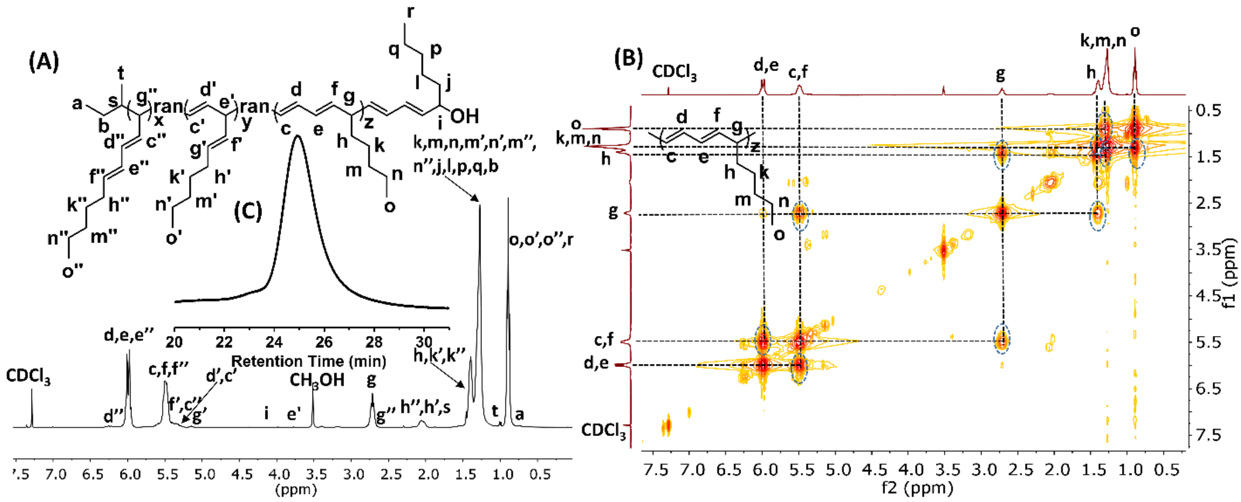

Figure S16. (A) ^{1}H NMR ($CDCl_3$, 25 °C, 400 MHz), (B) ^{1}H-^{1}H COSY (400-400 MHz, 25 °C, $CDCl_3$) spectra and (C) SEC (eluent, THF; flow rate, 1.0 ml min^{-1}; 25 °C) trace of C5-polymer (Table 1, Entry 14, [Ylide 4]$_0$/[*s*-Bu_3B]$_0$ = 210/1).

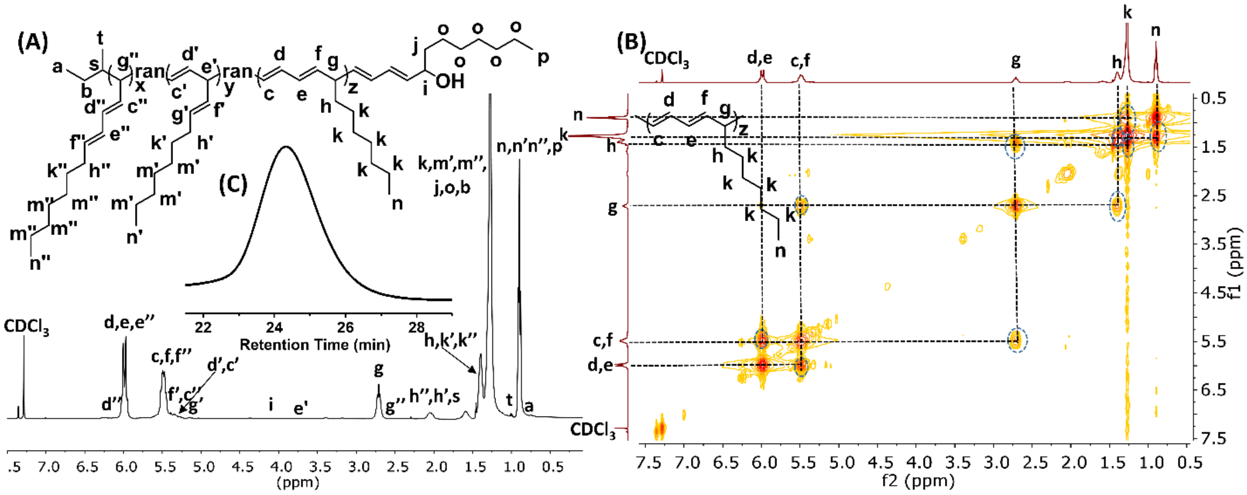

Figure S17. (A) ^{1}H NMR ($CDCl_3$, 25 °C, 400 MHz), (B) ^{1}H-^{1}H COSY (400-400 MHz, 25 °C, $CDCl_3$) spectra and (C) SEC (eluent, THF; flow rate, 1.0 ml min^{-1}; 25 °C) trace of C5-polymer (Table 1, Entry 15, [Ylide 5]$_0$/[*s*-Bu_3B]$_0$ = 210/1).

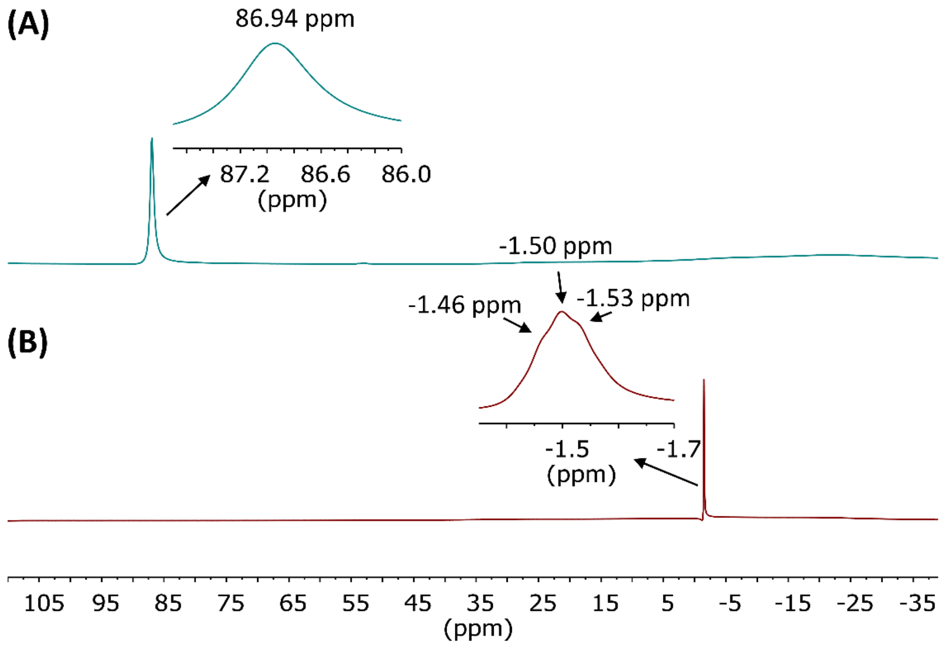

Figure S18. ^{11}B NMR spectra (128 MHz, 25 °C, $CDCl_3$) of (A) Et_3B and (B) mixture of Et_3B and Ylide 1 ($[Ylide\ 1]_0/[Et_3B]_0 = 1/1$).

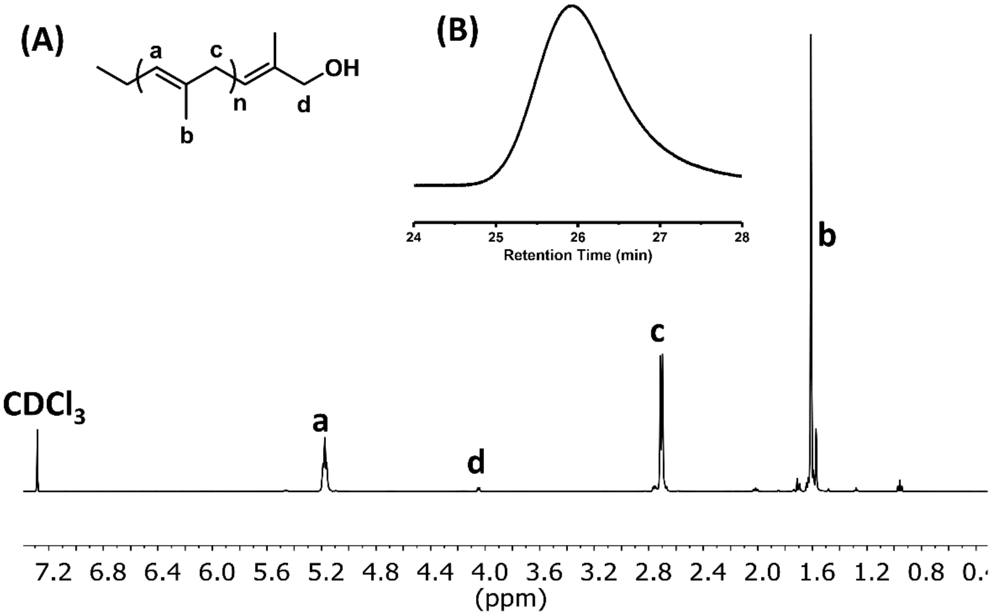

Figure S19. (A) ^1H NMR ($CDCl_3$, 25 °C, 400 MHz) and (B) SEC trace (eluent, THF; flow rate, 1.0 ml min^{-1}; 25 °C) of poly(2-methyl-propenylene synthesized by BEt_3-initiated C3 polymerization ($M_{n,NMR}$ = 4.3 kg mol^{-1}, $Đ$ = 1.07).

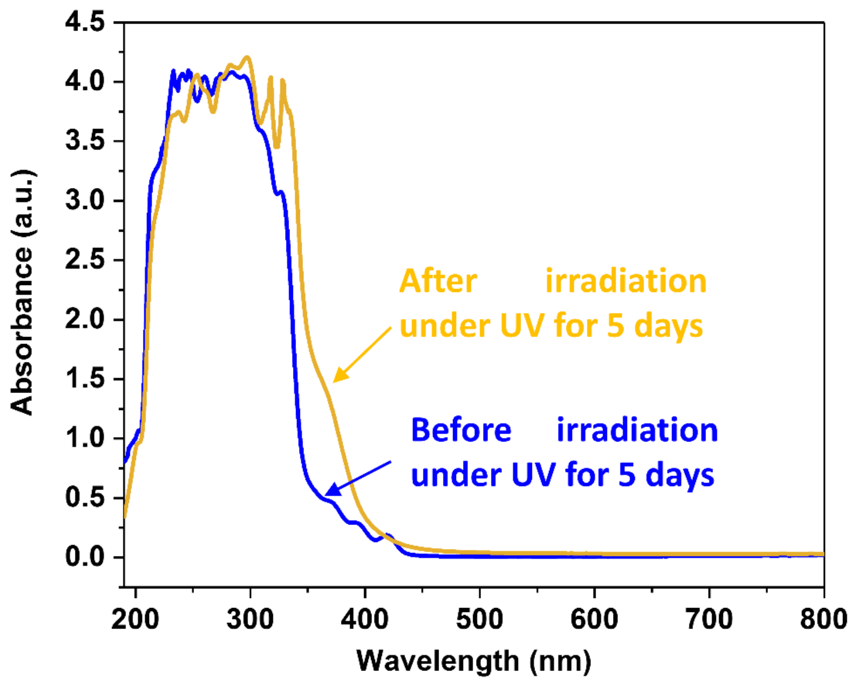

Figure S20. UV-vis spectra of MeDEY-6 before and after irradiation under 365 nm UV light for 5 days.

Computational Details

All computations were performed using the Gaussian 16[2] software package in a Linux multi-processor environment. Geometry optimizations and vibration analysis were performed by using the dispersion-corrected[3] BP86[4] (BP86+D3 (BJ)) density functional theory (DFT) method with the def2-TZVPP[5] basis set and the CPCM[6] (THF) solvent model. The enthalpies and free energies were obtained from the frequency calculations at 298.15 K and 1 atm. pressure. Normal modes of all structures were examined to verify that equilibrium structures possess no imaginary frequencies and that one imaginary frequency corresponding to the bond formation or the bond breaking was obtained for transition state structures. Intrinsic reaction coordinate (IRC) calculations were also performed to verify that transition state structures are shown to connect the reactant and product on the potential energy surfaces of reactions. To convert between Hartree's and kcal/mol, the 1 Hartree = 627.5 kcal/mol conversion factor was used.

Cartesian coordinates (Å) and energies (in Hartree) of the calculated structures

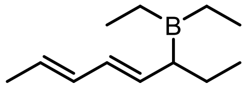

Sum of electronic and zero-point Energies= -495.861462

Sum of electronic and thermal Enthalpies= -495.842460

Sum of electronic and thermal Free Energies= -495.907849

B	-1.40542000	0.44327000	-0.23938100
C	-1.07974300	-1.11587500	-0.36540600
C	-2.11683400	0.95974300	1.07434200
C	0.25354600	-1.00862500	0.32133200
C	-1.04224000	1.40437400	-1.44574300
C	-2.09653200	2.45413300	1.42584900
H	-1.80868000	0.36119100	1.94875500
H	-3.17227000	0.64849900	0.91561200
C	1.44051900	-0.76944700	-0.28982600
H	0.23647400	-1.05762100	1.41685400
C	0.09923500	2.41428200	-1.19050000
H	-0.80461200	0.82332500	-2.35235300
H	-1.96044400	1.97646500	-1.68370900
H	-2.77073200	2.68468000	2.26449800
H	-1.08847600	2.78223800	1.71752900
H	-2.40833000	3.07488700	0.57292500
C	2.69600300	-0.58931500	0.40345400
H	1.46733000	-0.70735000	-1.38408300
H	-0.10519000	3.05324200	-0.32155900
H	0.25302900	3.07284200	-2.05866400
H	1.04474100	1.88955800	-0.99459800
C	3.87612400	-0.35045600	-0.20937600
H	2.66877100	-0.64858900	1.49837900
C	5.18274800	-0.15563800	0.49020200

H	3.88895000	-0.28997500	-1.30387200
H	5.92552200	-0.90222000	0.16344300
H	5.61781300	0.82998000	0.25548100
H	5.07362800	-0.23290200	1.58078200
H	-0.92096000	-1.38843000	-1.42221100
C	-2.03068500	-2.12178600	0.29875000
H	-1.55734100	-3.11835100	0.31688300
H	-2.18857200	-1.84149900	1.35299700
C	-3.37876300	-2.21015400	-0.42121900
H	-4.04480800	-2.94322500	0.05583500
H	-3.89512900	-1.23832800	-0.42216300
H	-3.24387600	-2.51218100	-1.47101500

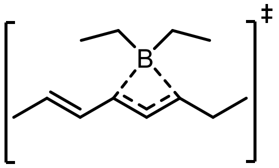

Sum of electronic and zero-point Energies= -495.844760

Sum of electronic and thermal Enthalpies= -495.826826

Sum of electronic and thermal Free Energies= -495.888900

B	-0.52305300	0.35968800	-0.05797400
C	-1.74662900	-1.10417000	-0.65741900
C	-0.62647300	0.79894200	1.48701400
C	-0.45859800	-1.40422200	-0.22629400
C	-0.84025500	1.47776900	-1.18139700
C	0.68425200	1.25669400	2.15624700
H	-1.06702800	-0.00368100	2.10254000
H	-1.35206600	1.63087700	1.54334500
C	0.71348600	-0.70363700	-0.69826200
H	-0.36470200	-1.88270600	0.75440200

C	0.18191200	2.62668100	-1.13701700
H	-0.85490500	1.05276600	-2.19763600
H	-1.84940100	1.89103200	-1.00933900
H	0.50587700	1.65714500	3.16646900
H	1.39777000	0.42694100	2.25146400
H	1.18563900	2.04178600	1.57225800
C	2.00532600	-0.91986300	-0.03838900
H	0.77525700	-0.47330100	-1.76541900
H	0.17290800	3.13525700	-0.16200500
H	-0.02709300	3.38732300	-1.90507500
H	1.20474200	2.25477500	-1.30249000
C	3.20331600	-0.62713600	-0.57462800
H	1.97331600	-1.34106900	0.97359900
C	4.51911400	-0.82046800	0.11598900
H	3.23465500	-0.20408400	-1.58567600
H	5.18369600	-1.48842700	-0.45643600
H	5.06069600	0.13418100	0.22390000
H	4.38570200	-1.25007800	1.11909100
H	-1.90837400	-0.76549700	-1.68220100
C	-2.97477900	-1.49135600	0.11802100
H	-3.43678100	-2.37599500	-0.35480500
H	-2.68666500	-1.79833200	1.13561400
C	-4.01012200	-0.35947600	0.18244600
H	-4.30986400	-0.04272000	-0.82726500
H	-4.91401800	-0.68116000	0.71813600
H	-3.59739900	0.51798000	0.69952400

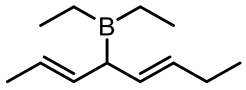

Sum of electronic and zero-point Energies= -495.853493

Sum of electronic and thermal Enthalpies= -495.834140

Sum of electronic and thermal Free Energies= -495.901515

B	-0.50741000	-1.08748300	0.15567300
C	2.37377800	-0.01031600	-0.17209500
C	-1.01011700	-1.37231300	1.62394800
C	1.22449900	0.42464500	0.37374000
C	-0.42901100	-2.24043600	-0.92312500
C	-2.54121900	-1.61421700	1.58368500
H	-0.79605800	-0.55097200	2.32554100
H	-0.52631400	-2.27767200	2.02845200
C	-0.12768400	0.39562200	-0.30384700
H	1.23114300	0.75687600	1.41837300
C	-1.51861500	-2.06438000	-2.00727500
H	0.55843800	-2.20942200	-1.41619500
H	-0.52026200	-3.24029100	-0.46780800
H	-2.93917800	-1.82096400	2.58779000
H	-3.07233500	-0.73570300	1.18821600
H	-2.79527800	-2.47058200	0.94222600
C	-1.07657200	1.45235400	0.16894300
H	-0.00340300	0.44937500	-1.39661000
H	-2.52837100	-2.11776800	-1.57463700
H	-1.44812300	-2.84392900	-2.77990000
H	-1.42948100	-1.08947100	-2.50852100
C	-1.84699600	2.21018400	-0.62495800
H	-1.16046000	1.57734600	1.25585700
C	-2.83856000	3.22782200	-0.14334600
H	-1.76233800	2.08399700	-1.71125400
H	-2.61323600	4.23072100	-0.54133000

H	-3.86006000	2.98411500	-0.47925100
H	-2.84768900	3.28956800	0.95421900
H	2.36673300	-0.35218500	-1.21470500
C	3.70257400	-0.03477400	0.52358100
H	3.57632700	0.28214600	1.57072400
H	4.07885600	-1.07280400	0.55097000
C	4.75065700	0.84906400	-0.17390600
H	4.43575700	1.90247900	-0.17380400
H	5.72431100	0.78165900	0.33184300
H	4.89167700	0.54137800	-1.22062700

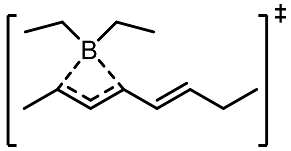

Sum of electronic and zero-point Energies= -495.843735

Sum of electronic and thermal Enthalpies= -495.825743

Sum of electronic and thermal Free Energies= -495.888369

B	-1.36638600	0.16723200	-0.04951300
C	-2.27128700	-1.45052300	-0.54390000
C	-1.55268200	0.63692500	1.47842100
C	-0.95006800	-1.54423100	-0.08634300
C	-1.75474900	1.18075500	-1.25537800
C	-0.97701800	2.01830900	1.83731700
H	-1.13003500	-0.10396500	2.17757300
H	-2.63714800	0.64737500	1.69738600
C	0.09855600	-0.69945200	-0.57992100
H	-0.81409600	-1.93351000	0.92776300
C	-0.70311800	2.26497600	-1.55053900
H	-1.95055400	0.63806800	-2.19323900

H	-2.71169300	1.66576100	-0.98975600
H	-1.10971100	2.24745400	2.90613200
H	0.09998400	2.07259900	1.61775000
H	-1.46786700	2.82165700	1.26954600
C	1.34464000	-0.55355900	0.17247800
H	0.16530200	-0.52231000	-1.65619900
H	-0.48642500	2.88528800	-0.67143900
H	-1.03735200	2.93710800	-2.35618700
H	0.24892500	1.81516100	-1.86997900
C	2.50600100	-0.09944800	-0.33303200
H	1.29948800	-0.80703800	1.23906300
C	3.77249600	0.08146400	0.45033900
H	2.55265800	0.15480600	-1.39952300
H	4.07857200	1.14250500	0.41590700
H	3.58597200	-0.15548700	1.50997400
C	4.93086700	-0.77837100	-0.08408800
H	5.13327800	-0.55221700	-1.14162300
H	5.85540800	-0.59628800	0.48251800
H	4.68886000	-1.84860700	-0.01156600
H	-2.45870800	-1.26345400	-1.60147500
C	-3.42274600	-1.99894000	0.24980100
H	-4.28458400	-1.31559200	0.21467200
H	-3.76348000	-2.96549300	-0.15446100
H	-3.15022300	-2.14741700	1.30379500

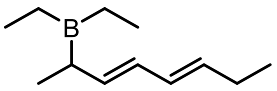

Sum of electronic and zero-point Energies= -495.855856

Sum of electronic and thermal Enthalpies= -495.836519

Sum of electronic and thermal Free Energies= -495.904978

B	1.98090300	0.09464700	-0.34886200
C	-3.36561600	0.31977600	-0.30115800
C	1.75526100	1.65832800	-0.28472600
C	-2.41310500	-0.07630100	0.56924600
C	2.82197500	-0.51366900	-1.53428800
C	3.08146700	2.42348300	-0.06334200
H	1.02679500	1.94582500	0.48916500
H	1.32667500	1.98586500	-1.25043300
C	-1.01117100	-0.23840500	0.23989900
H	-2.70124200	-0.29134900	1.60605000
C	3.71895800	-1.72908100	-1.24455200
H	2.02309700	-0.82484700	-2.24436600
H	3.39084500	0.25833100	-2.07826800
H	2.92354100	3.51191700	-0.07178100
H	3.53508700	2.16431800	0.90470300
H	3.81909500	2.18773000	-0.84395500
C	-0.06093200	-0.63390000	1.11430500
H	-0.72840500	-0.01834800	-0.79733400
H	4.55028200	-1.45459800	-0.57858300
H	4.15742900	-2.14282400	-2.16442700
H	3.16096300	-2.53917500	-0.75302700
C	1.39311800	-0.84486100	0.79236500
H	-0.37141000	-0.84545300	2.14708800
C	2.27961600	-0.59427800	2.04324600
H	1.52439700	-1.91054900	0.52371600
H	1.97247500	-1.25174700	2.87119800
H	3.34141700	-0.79268300	1.83679000
H	2.18991900	0.44588000	2.38880800

H	-3.07273700	0.52800200	-1.33812000
C	-4.81965900	0.47976300	0.02343800
H	-4.98124900	0.29404000	1.09697300
H	-5.12466800	1.52458400	-0.16556200
C	-5.71812100	-0.44895100	-0.81282400
H	-5.48435500	-1.50422000	-0.61137000
H	-6.78035400	-0.28252900	-0.58367600
H	-5.57286100	-0.27282600	-1.88893100

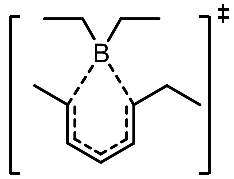

Sum of electronic and zero-point Energies= -495.780478

Sum of electronic and thermal Enthalpies= -495.763200

Sum of electronic and thermal Free Energies= -495.822127

C	-1.57945400	-1.29537100	-0.19676500
C	-0.68493400	-2.19433800	0.43577900
C	0.49845500	-2.62651500	-0.25291200
H	-0.73864800	-2.26931500	1.52854000
C	1.22559300	-1.56311100	0.30713800
H	0.44011500	-2.64944600	-1.34813900
C	1.30659800	-0.30299200	-0.41202200
H	1.34781100	-1.50638000	1.39741300
C	2.40499900	0.66572300	0.02684700
H	1.32196300	-0.46085100	-1.50291200
H	2.19757100	1.66138600	-0.39426200
H	2.37465900	0.77680000	1.12226300
C	3.80135700	0.20723500	-0.40777300
H	3.85818700	0.10193900	-1.50170500

H	4.57589500	0.92305900	-0.09635100
H	4.04572400	-0.77169000	0.03242000
H	-1.59102400	-1.34506900	-1.29339600
C	-2.92621400	-1.04259700	0.45327000
H	-3.67361300	-1.78273400	0.12682500
H	-2.85049600	-1.10859100	1.54803700
H	-3.31819800	-0.04885000	0.20390800
B	-0.31428200	0.29887500	-0.08706600
C	-0.71750400	1.17449800	-1.38761900
H	-0.54227700	0.59530600	-2.31068600
H	0.02638600	1.99466000	-1.42107200
C	-0.55142600	0.93968000	1.37112800
H	-1.63051000	0.90182800	1.59700700
H	-0.07577000	0.33255200	2.16200000
C	-2.12494900	1.78582000	-1.43543600
H	-2.38442500	2.29412500	-0.49464900
H	-2.21870400	2.52591400	-2.24565700
H	-2.89064200	1.01728300	-1.61305900
C	-0.10628200	2.40781900	1.53375400
H	0.97809900	2.53026500	1.41068200
H	-0.58959300	3.05605000	0.78775200
H	-0.36853300	2.80228900	2.52801900

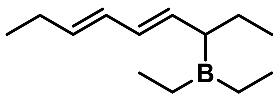

Sum of electronic and thermal Free Energies= -535.211783

B	-1.91305100	0.37731300	-0.23631800
C	-1.45794300	-1.14875100	-0.36842900
C	-2.66085000	0.82659200	1.08198700

C	-0.13748200	-0.92970800	0.31605700
C	-1.63689900	1.36799500	-1.44175900
C	-2.77299900	2.31632700	1.43529000
H	-2.29340300	0.25774100	1.95334800
H	-3.68464600	0.42062200	0.93058900
C	1.02170900	-0.58070200	-0.29565400
H	-0.14684700	-0.98811800	1.41120800
C	-0.59375500	2.47923700	-1.18713000
H	-1.34678800	0.81108200	-2.34823400
H	-2.60416800	1.85252700	-1.68029000
H	-3.45865600	2.48458400	2.27943500
H	-1.79631200	2.73379300	1.71933200
H	-3.14617900	2.90674500	0.58550100
C	2.25777600	-0.29517700	0.39715500
H	1.04009900	-0.50766600	-1.38933300
H	-0.85692600	3.09813400	-0.31952000
H	-0.50040600	3.14772800	-2.05630200
H	0.39604900	2.04432700	-0.98982800
C	3.41055700	0.05627300	-0.21327400
H	2.23849500	-0.36696100	1.49157800
C	4.70299300	0.34641800	0.48486200
H	3.42220900	0.12370100	-1.30840000
H	5.01673000	1.38081400	0.25701500
H	4.55505500	0.29449800	1.57489100
C	5.83140700	-0.61016200	0.05889400
H	5.99628300	-0.56531400	-1.02771200
H	6.77687100	-0.34975000	0.55543300
H	5.58173000	-1.64899200	0.31794700
H	-1.27920000	-1.40342600	-1.42648600

C	-2.31960000	-2.23265400	0.29467500
H	-1.76446800	-3.18611700	0.30934600
H	-2.49872900	-1.96910400	1.34988900
C	-3.65684500	-2.43143200	-0.42351300
H	-4.25853300	-3.21890700	0.05232200
H	-4.25241000	-1.50600300	-0.42067500
H	-3.49901300	-2.71803700	-1.47443000

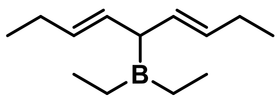

Sum of electronic and thermal Free Energies= -535.206302

B	0.11978700	1.36957300	-0.17852600
C	2.52646400	-0.58009400	0.06944600
C	-0.37910100	1.83057100	-1.60746300
C	1.29375300	-0.58159900	-0.46671900
C	0.69370800	2.46293500	0.80737800
C	-1.85742600	2.28433700	-1.49185700
H	-0.31668100	1.02713400	-2.35862800
H	0.21922900	2.67462700	-1.98938200
C	0.02195500	-0.16687600	0.24607200
H	1.18405900	-0.83414900	-1.52787000
C	0.91652500	2.10782200	2.28260000
H	1.63501600	2.83341000	0.35782800
H	0.01973900	3.33760100	0.72994400
H	-2.24876700	2.62201500	-2.46266400
H	-2.49873100	1.46389000	-1.13825900
H	-1.96670400	3.11748000	-0.78182200
C	-1.19627400	-0.89127400	-0.23496300
H	0.14119700	-0.29277900	1.33217400

H	-0.01356900	1.75606000	2.75356600
H	1.27464800	2.97103400	2.86312600
H	1.65973700	1.30575200	2.39858600
C	-2.13640600	-1.43378800	0.55197000
H	-1.32811900	-0.94956500	-1.32283400
C	-3.39541200	-2.09099000	0.06208400
H	-2.01199200	-1.37403100	1.64109300
H	-3.44837300	-3.12520900	0.44536200
H	-3.36681700	-2.16353500	-1.03691900
C	-4.66242800	-1.33791700	0.50271700
H	-4.67339100	-0.31887900	0.08945000
H	-5.57193700	-1.85529900	0.16458100
H	-4.70966500	-1.25436800	1.59881900
H	2.64307100	-0.32494400	1.12974600
C	3.78417800	-0.93727100	-0.66536800
H	3.55018900	-1.13381900	-1.72344800
H	4.47028400	-0.07172200	-0.64940400
C	4.50574800	-2.14733400	-0.04834600
H	3.87273500	-3.04511300	-0.09453500
H	5.44402000	-2.36115800	-0.57955800
H	4.75018400	-1.96343100	1.00835800

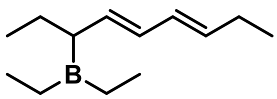

Sum of electronic and thermal Free Energies= -535.212736

B	1.71876300	0.58239100	0.26441100
C	-3.57619400	0.27819400	-0.03198300
C	2.21704100	0.47254500	1.76711700
C	-2.43670100	-0.31217800	0.39026200

C	1.60405800	2.02728900	-0.37114500
C	3.76193700	0.55534300	1.84171700
H	1.89409300	-0.46324200	2.25092800
H	1.79887500	1.29896200	2.36756600
C	-1.17477500	-0.24710900	-0.31162500
H	-2.45064900	-0.87901400	1.32933400
C	1.37995500	2.16487800	-1.88237900
H	0.80961000	2.56466800	0.18131700
H	2.52262300	2.57231500	-0.08037900
H	4.11239400	0.55005400	2.88429700
H	4.23541700	-0.29493900	1.33047300
H	4.14025700	1.47428300	1.36989900
C	-0.03202400	-0.84251700	0.11160800
H	-1.16129900	0.32162400	-1.24857600
H	2.16995300	1.65184200	-2.45121100
H	1.37179600	3.21726200	-2.20354600
H	0.42208900	1.72238300	-2.19266400
C	1.31394900	-0.72579300	-0.54967900
H	-0.05844900	-1.39663400	1.05727300
C	2.19175500	-1.97490700	-0.38433700
H	1.17701100	-0.50592800	-1.62030500
H	1.63883700	-2.86453500	-0.73124900
H	2.39980600	-2.14204100	0.68539500
C	3.50603500	-1.85920100	-1.16064800
H	4.13348600	-2.75233700	-1.03000100
H	3.31496700	-1.73684500	-2.23757500
H	4.08886300	-0.98619400	-0.83044000
H	-3.55549700	0.84013000	-0.97420000
C	-4.89469000	0.21308000	0.67470900

H	-4.77958200	-0.33211400	1.62457900
H	-5.22046200	1.23617000	0.93444300
C	-5.99149900	-0.44478100	-0.18206400
H	-5.73088300	-1.48636400	-0.41825800
H	-6.95644300	-0.44279700	0.34439000
H	-6.12370600	0.09228500	-1.13285200

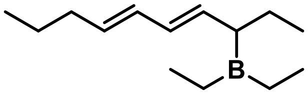

Sum of electronic and thermal Free Energies= -574.516787

B	2.42963900	-0.36603200	-0.20623900
C	1.96352100	1.15412700	-0.36584100
C	3.13711900	-0.79686100	1.14021200
C	0.62201600	0.92375200	0.27257400
C	2.20414600	-1.36813300	-1.41283400
C	3.25568700	-2.28242200	1.50873800
H	2.73232000	-0.22663700	1.99397900
H	4.16033600	-0.37877900	1.02159100
C	-0.50926200	0.55334600	-0.37776500
H	0.59081100	0.99180500	1.36674900
C	1.16914300	-2.49230600	-1.18363000
H	1.93459300	-0.82080200	-2.33143400
H	3.18505900	-1.84024700	-1.61845000
H	3.91412500	-2.43557300	2.37709700
H	2.27519600	-2.71021700	1.76270200
H	3.66514200	-2.87440300	0.67692700
C	-1.76588600	0.25624300	0.27152600
H	-0.48635100	0.47048800	-1.47064600
H	1.41418500	-3.10298800	-0.30498600

H	1.11131600	-3.16658400	-2.05142700
H	0.16797100	-2.06997600	-1.01891000
C	-2.89000400	-0.11785500	-0.37799000
H	-1.78836800	0.33774300	1.36518300
C	-4.20287900	-0.41620500	0.27405300
H	-2.85871200	-0.19581600	-1.47190400
H	-4.51237500	-1.44881000	0.02778200
H	-4.09581000	-0.37131800	1.37024700
C	-5.32710900	0.53840800	-0.17502800
H	-5.40797500	0.50723300	-1.27402400
H	-5.04179400	1.57066600	0.08424300
C	-6.67745100	0.19280000	0.45643900
H	-7.46424000	0.88628400	0.12720300
H	-6.99153600	-0.82631100	0.18419000
H	-6.62458300	0.24197600	1.55462700
H	1.81991900	1.39944800	-1.43142900
C	2.78846300	2.25244000	0.31970400
H	2.22249900	3.19956200	0.30738300
H	2.93249100	1.99845800	1.38262500
C	4.14833400	2.46159600	-0.35149000
H	4.72357000	3.25934900	0.13969800
H	4.75381900	1.54315700	-0.32029500
H	4.02517300	2.73887700	-1.40952300

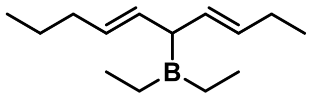

Sum of electronic and thermal Free Energies= -574.511773

B	0.73150300	1.41712500	-0.18416400
C	2.87336100	-0.81377400	0.06464500

C	0.28488000	1.92839800	-1.61309800
C	1.64451000	-0.67204500	-0.46136100
C	1.44887500	2.43452700	0.78926700
C	-1.12290100	2.56723600	-1.48976800
H	0.23875900	1.11933600	-2.35932000
H	0.98255100	2.68753500	-2.00454900
C	0.44111000	-0.09118300	0.25401800
H	1.49368100	-0.92594500	-1.51714500
C	1.61950100	2.07026100	2.26926100
H	2.43310800	2.66360200	0.33717000
H	0.90383800	3.39354000	0.69913500
H	-1.47454100	2.94914000	-2.45936600
H	-1.86095100	1.83616100	-1.12889500
H	-1.12050900	3.40968600	-0.78224900
C	-0.86371400	-0.65367500	-0.21569700
H	0.54927100	-0.22183700	1.34073700
H	0.64919100	1.85706300	2.74222200
H	2.09381100	2.88236300	2.84012400
H	2.24373000	1.17405300	2.39706900
C	-1.86783600	-1.05225200	0.57843400
H	-1.00413000	-0.70922500	-1.30266100
C	-3.20552000	-1.53244100	0.09534300
H	-1.73460900	-0.99483800	1.66645500
H	-3.42007700	-2.53379800	0.51127600
H	-3.18467800	-1.64460000	-1.00163100
C	-4.35784100	-0.58975300	0.48998100
H	-4.16482500	0.40564700	0.05772000
H	-4.35456700	-0.45518600	1.58447600
C	-5.72540600	-1.10315100	0.03373100

H	-5.94677200	-2.08566300	0.47792000
H	-6.53235200	-0.41467700	0.32276100
H	-5.75893600	-1.21885400	-1.06037600
H	3.03119100	-0.55661700	1.11910400
C	4.06968200	-1.33702500	-0.67326500
H	3.80321700	-1.51891900	-1.72626400
H	4.85914100	-0.56445900	-0.67638700
C	4.63918200	-2.61865900	-0.04175600
H	3.89797800	-3.43026200	-0.06874700
H	5.53822200	-2.95686300	-0.57617200
H	4.91444900	-2.45079100	1.00999800

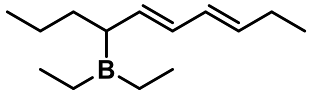

Sum of electronic and thermal Free Energies= -574.518199

B	1.30209400	1.00516400	0.20552300
C	-3.92808600	0.05404400	-0.09611400
C	1.77664100	1.19816600	1.70739700
C	-2.74418700	-0.32323700	0.43426200
C	1.05208600	2.30408200	-0.66272700
C	3.30763700	1.42453600	1.77337400
H	1.52418900	0.33451200	2.34336300
H	1.27933100	2.07624400	2.15442500
C	-1.47554400	-0.23034500	-0.25230300
H	-2.72515100	-0.72546000	1.45467100
C	0.85986400	2.16515100	-2.17826300
H	0.18677900	2.83098800	-0.21657200
H	1.89593200	2.98976300	-0.45517500
H	3.63602100	1.63718700	2.80135200

H	3.86028000	0.53970200	1.42679200
H	3.61781200	2.27236400	1.14458500
C	-0.28767300	-0.61388700	0.27773800
H	-1.49544100	0.17484800	-1.27062000
H	1.71780900	1.65720500	-2.64392600
H	0.74730300	3.14263400	-2.67077800
H	-0.03445900	1.57298000	-2.42162000
C	1.05548600	-0.45538500	-0.38022200
H	-0.28091500	-1.00437000	1.30220100
C	2.06247400	-1.54871700	0.00064500
H	0.92446400	-0.43240700	-1.47361700
H	1.62927600	-2.54582900	-0.19433200
H	2.26288700	-1.50956300	1.08529300
C	3.38085900	-1.41688400	-0.76829800
H	3.17643200	-1.50698000	-1.84841600
H	3.78595200	-0.40116700	-0.62326800
C	4.42418600	-2.45390600	-0.34882300
H	4.04903500	-3.47717600	-0.50358200
H	5.35573000	-2.34843000	-0.92319400
H	4.67421100	-2.35102400	0.71828800
H	-3.93937100	0.45136400	-1.11887400
C	-5.25358400	-0.04522500	0.59329500
H	-5.10975200	-0.41015100	1.62231300
H	-5.69943700	0.96224200	0.67418600
C	-6.24188700	-0.95652700	-0.15686200
H	-5.85969700	-1.98584000	-0.21214800
H	-7.21795000	-0.97892600	0.34818600
H	-6.40136300	-0.60171600	-1.18574500

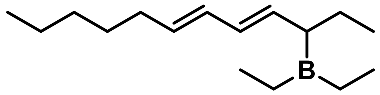

Sum of electronic and thermal Free Energies= -653.127148

B	3.21719800	-0.06541400	-0.21283700
C	2.33342500	1.24864600	-0.42499700
C	4.03033900	-0.21491900	1.13452900
C	1.11852600	0.67520900	0.25023900
C	3.27700300	-1.14569100	-1.37059500
C	4.56737200	-1.58760000	1.56364700
H	3.49025100	0.25884000	1.97223300
H	4.89282400	0.46774500	0.97346300
C	0.12993900	-0.02322200	-0.36191900
H	1.08073800	0.77662200	1.34162800
C	2.60048600	-2.50237100	-1.07132400
H	2.85866700	-0.74037200	-2.30694200
H	4.34962200	-1.33361600	-1.57394000
H	5.25270600	-1.50729700	2.42091100
H	3.75047000	-2.26099100	1.86047600
H	5.11695600	-2.08020100	0.74799300
C	-0.98868600	-0.62961900	0.32328400
H	0.16358700	-0.14030600	-1.45144500
H	3.01142600	-2.97570900	-0.17011800
H	2.73042600	-3.20767100	-1.90603400
H	1.52184100	-2.36988700	-0.90781200
C	-1.97535600	-1.31865100	-0.29028300
H	-1.02214800	-0.51476600	1.41352700
C	-3.15109500	-1.94246000	0.39411100
H	-1.93691900	-1.42335700	-1.38201000
H	-3.15685700	-3.02832800	0.19284400

H	-3.05469900	-1.82048400	1.48547800
C	-4.50025100	-1.35683300	-0.07476700
H	-4.59016800	-1.48555000	-1.16722400
H	-5.31776000	-1.94429900	0.37463400
C	-4.67628300	0.12092300	0.28125500
H	-4.57745500	0.24492300	1.37451500
H	-3.85395200	0.70580000	-0.16471800
C	-6.01785700	0.70037500	-0.17616400
H	-6.83860200	0.11497700	0.27222400
H	-6.11314200	0.57146900	-1.26776000
C	-6.18366500	2.17914600	0.18371400
H	-5.39282700	2.78890000	-0.27940100
H	-7.15248000	2.57366200	-0.15493900
H	-6.12250200	2.32977100	1.27244500
H	2.11656200	1.39397800	-1.49652000
C	2.81405700	2.56751800	0.19681800
H	1.99906700	3.31040100	0.15873900
H	3.03533700	2.41412700	1.26570900
C	4.04923700	3.12840700	-0.51263200
H	4.37492600	4.07907600	-0.06682500
H	4.89424000	2.42545100	-0.45858400
H	3.84072800	3.31100900	-1.57784900

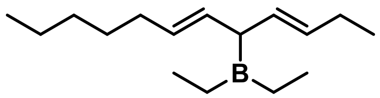

Sum of electronic and thermal Free Energies= -653.121338

B	1.70375400	1.41462300	-0.19751400
C	3.78698200	-0.87576300	0.08150700
C	1.27660700	1.92881700	-1.63130500

C	2.56766900	-0.69960400	-0.45613700
C	2.44057600	2.41962400	0.77409300
C	-0.11566000	2.60266600	-1.51805000
H	1.21393200	1.11686600	-2.37316300
H	1.99444300	2.66838800	-2.02373900
C	1.37448300	-0.08390800	0.24682000
H	2.41944200	-0.95134000	-1.51283400
C	2.59919300	2.05774300	2.25597700
H	3.43077600	2.62546100	0.32381600
H	1.91687800	3.38997800	0.67859600
H	-0.45420600	2.98606800	-2.49171000
H	-0.87269600	1.89245200	-1.15487000
H	-0.09529400	3.44983400	-0.81650000
C	0.05929900	-0.61661100	-0.22908100
H	1.47164700	-0.21053800	1.33506500
H	1.62325300	1.86730300	2.72705600
H	3.08922100	2.86179800	2.82495700
H	3.20376700	1.14892300	2.38897400
C	-0.96208000	-0.98046600	0.55961900
H	-0.07316500	-0.67898700	-1.31668500
C	-2.30816400	-1.42791100	0.06835400
H	-0.83745800	-0.91554200	1.64822500
H	-2.55676700	-2.41613000	0.49606200
H	-2.27808400	-1.55618700	-1.02648700
C	-3.43398400	-0.44463400	0.43654300
H	-3.20738200	0.53971500	-0.00720100
H	-3.44224700	-0.29396600	1.52996600
C	-4.81677400	-0.91115600	-0.02488000
H	-5.03681900	-1.89743100	0.42073800

H	-4.80321700	-1.06711900	-1.11813400
C	-5.94029600	0.06708100	0.33154800
H	-5.95183100	0.22133700	1.42388200
H	-5.71627500	1.05167900	-0.11283700
C	-7.31873100	-0.40726600	-0.13613000
H	-7.57851700	-1.37546600	0.31869600
H	-8.10749600	0.31056800	0.13130200
H	-7.34147600	-0.53838700	-1.22886000
H	3.94232100	-0.62188000	1.13713500
C	4.97472100	-1.43372100	-0.64475000
H	4.71254300	-1.61035200	-1.69972600
H	5.78534500	-0.68337600	-0.64242000
C	5.50290600	-2.72929200	-0.00583900
H	4.73973100	-3.52009200	-0.03776800
H	6.39685600	-3.09344500	-0.53166900
H	5.77352500	-2.56684100	1.04797500

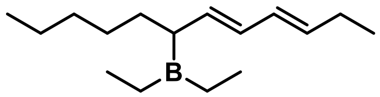

Sum of electronic and thermal Free Energies= -653.127976

B	-0.27514400	1.44783700	-0.17269400
C	4.71123900	-0.43229100	0.17027000
C	-0.64289900	1.87513700	-1.65591000
C	3.50674000	-0.53260500	-0.43330200
C	0.14074100	2.58702800	0.84302700
C	-2.11738100	2.34252900	-1.74198400
H	-0.49416700	1.05696100	-2.37886800
H	0.00204700	2.70854700	-1.98389800
C	2.24118000	-0.29338400	0.22316600

H	3.46766100	-0.81022000	-1.49387900
C	0.23962000	2.25641500	2.33763000
H	1.09907800	3.00806000	0.48259900
H	-0.57104100	3.42112000	0.69115700
H	-2.35887800	2.71879800	-2.74690600
H	-2.81308600	1.52063300	-1.52106800
H	-2.32702600	3.15141900	-1.02631000
C	1.03254800	-0.40320500	-0.38176700
H	2.28072700	-0.01202500	1.28189500
H	-0.70879200	1.84930800	2.71933400
H	0.48524600	3.14337100	2.94068400
H	1.01548700	1.50251900	2.53597600
C	-0.29457300	-0.08757200	0.25096800
H	1.00935800	-0.67225900	-1.44440000
C	-1.45179100	-0.94496300	-0.27894700
H	-0.21318300	-0.20336300	1.34331600
H	-1.20424800	-2.01667900	-0.18152500
H	-1.58743900	-0.75933100	-1.35833200
C	-2.76398500	-0.66249100	0.45813800
H	-2.64284500	-0.91960100	1.52491100
H	-2.97160700	0.42170000	0.43309800
C	-3.96236500	-1.41916400	-0.11720000
H	-3.75454900	-2.50372300	-0.09934000
H	-4.08391100	-1.15043800	-1.18168600
C	-5.27304500	-1.14247000	0.62558200
H	-5.15049100	-1.41395200	1.68785400
H	-5.47382600	-0.05771000	0.60967000
C	-6.46783200	-1.89595400	0.03559300
H	-6.30386600	-2.98399700	0.06824000

H	-7.39560000	-1.68069200	0.58502000
H	-6.62946300	-1.61730400	-1.01703700
H	4.74237800	-0.15879600	1.23245800
C	6.03075800	-0.68923800	-0.48935100
H	5.87495500	-0.90528600	-1.55791400
H	6.64896900	0.22480800	-0.43808800
C	6.80764300	-1.84075900	0.17423700
H	6.24942100	-2.78467200	0.09649100
H	7.78797500	-1.98086600	-0.30284900
H	6.97684500	-1.63759900	1.24195100

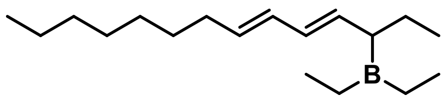

Sum of electronic and thermal Free Energies= -731.736227

B	4.06132200	0.26603200	-0.21277100
C	2.95524400	1.39741400	-0.43482900
C	4.88352300	0.27362400	1.13740300
C	1.86197900	0.61762500	0.24142500
C	4.31903000	-0.79216400	-1.36373000
C	5.65940800	-0.97677500	1.57469600
H	4.26316400	0.64495400	1.97118500
H	5.60795700	1.10114400	0.97541600
C	1.01646300	-0.25013800	-0.36842700
H	1.80407100	0.71539200	1.33225800
C	3.89569900	-2.24664500	-1.05855400
H	3.83846800	-0.47354000	-2.30371800
H	5.40876500	-0.78565100	-1.56269000
H	6.31444900	-0.77018300	2.43450900
H	4.97728500	-1.78668600	1.87074600

H	6.29351200	-1.36368900	0.76339500
C	0.02484300	-1.04537100	0.31918500
H	1.07221400	-0.36397800	-1.45738600
H	4.38279900	-2.63470200	-0.15454100
H	4.15164000	-2.92110400	-1.88961800
H	2.81043500	-2.30864600	-0.89699100
C	-0.82270700	-1.90185000	-0.29146500
H	-0.02822700	-0.93525100	1.40914300
C	-1.86814000	-2.72299500	0.39607100
H	-0.76686300	-2.00083300	-1.38294800
H	-1.68739600	-3.79252400	0.18886800
H	-1.78730800	-2.59080100	1.48755100
C	-3.30132200	-2.37658900	-0.06175300
H	-3.37448600	-2.51126300	-1.15470400
H	-4.00220600	-3.09950400	0.38712100
C	-3.72699200	-0.95379700	0.30699800
H	-3.64598800	-0.82400700	1.40090500
H	-3.01832100	-0.23392000	-0.13615900
C	-5.14913900	-0.60915900	-0.14217800
H	-5.85960200	-1.32653900	0.30535900
H	-5.22845700	-0.74404300	-1.23540100
C	-5.57224200	0.81625200	0.22270800
H	-4.86130600	1.53306500	-0.22511100
H	-5.49238900	0.95192200	1.31590500
C	-6.99377500	1.16804700	-0.22676400
H	-7.70237900	0.45111600	0.22177200
H	-7.07173800	1.03046900	-1.31869400
C	-7.40343700	2.59609900	0.14254300
H	-6.72857600	3.33259300	-0.31999900

H	-8.42633300	2.82444700	-0.18994300
H	-7.36321100	2.74920600	1.23191900
H	2.71890500	1.49538200	-1.50762800
C	3.18794800	2.78474700	0.18028900
H	2.25262700	3.36842100	0.13529600
H	3.42911500	2.67956800	1.25069900
C	4.30479700	3.55507100	-0.52882800
H	4.45128800	4.55174300	-0.08861800
H	5.26263800	3.01670800	-0.46675100
H	4.07176000	3.69020300	-1.59603300

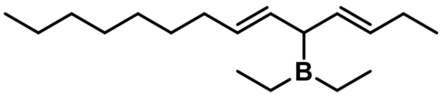

Sum of electronic and thermal Free Energies= -731.730771

B	2.67412400	1.41403600	-0.21002500
C	4.81127100	-0.82432200	0.07692300
C	2.23042200	1.90699400	-1.64628400
C	3.58563500	-0.68219300	-0.45638300
C	3.38993800	2.44363000	0.75140800
C	0.82228500	2.54724100	-1.53427900
H	2.18595300	1.08826600	-2.38201100
H	2.92885200	2.66107100	-2.04607400
C	2.38161800	-0.08847700	0.24681800
H	3.43865100	-0.94655800	-1.51017200
C	3.56195500	2.09685700	2.23540400
H	4.37354200	2.66927400	0.29616700
H	2.84314200	3.40058200	0.65029900
H	0.47236500	2.91574400	-2.50967200
H	0.08371000	1.82108700	-1.16463500

H	0.82332000	3.39933500	-0.83839900
C	1.07631500	-0.65254400	-0.21990900
H	2.48569300	-0.20458700	1.33561500
H	2.59236100	1.88690500	2.71126700
H	4.03467600	2.91654900	2.79665700
H	4.18832700	1.20362100	2.37301200
C	0.06437800	-1.02889800	0.57509800
H	0.94166500	-0.72672000	-1.30649800
C	-1.27519100	-1.50344000	0.09151200
H	0.19080200	-0.95208600	1.66271600
H	-1.50740100	-2.49102800	0.52970300
H	-1.24534100	-1.64210900	-1.00207900
C	-2.41526100	-0.53383900	0.45217400
H	-2.20295900	0.45010000	0.00053100
H	-2.42554500	-0.37448400	1.54433100
C	-3.79143000	-1.02348200	-0.00535100
H	-3.99685800	-2.00933900	0.44770800
H	-3.77506300	-1.18709900	-1.09729100
C	-4.92718400	-0.05806600	0.34439500
H	-4.94506200	0.10289100	1.43674500
H	-4.71786600	0.92850000	-0.10541700
C	-6.30419800	-0.54206800	-0.11795000
H	-6.51393700	-1.52930600	0.33063700
H	-6.28673400	-0.70216000	-1.21061600
C	-7.44125200	0.42279900	0.23224000
H	-7.45714200	0.58102300	1.32401400
H	-7.22882700	1.40869400	-0.21515600
C	-8.81269600	-0.07038100	-0.23633400
H	-9.06116800	-1.04024100	0.22134600

H	-9.61117900	0.63821300	0.02697300
H	-8.83170900	-0.20569200	-1.32865200
H	4.96526100	-0.55728100	1.12950500
C	6.00860200	-1.36053600	-0.65001100
H	5.74618500	-1.55160500	-1.70241500
H	6.80171700	-0.59173800	-0.65713300
C	6.56916200	-2.63849900	-0.00330100
H	7.46906600	-2.98614000	-0.53016600
H	6.84037600	-2.46161600	1.04803600
H	5.82430300	-3.44689800	-0.02576000

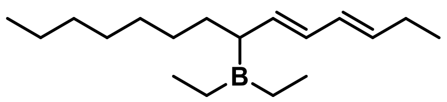

Sum of electronic and thermal Free Energies= -731.737548

B	0.81342400	1.59198600	-0.15915700
C	5.58299600	-0.76197400	0.20504300
C	0.52016800	2.09986500	-1.63363600
C	4.38627800	-0.73367900	-0.42134300
C	1.32123500	2.65185000	0.89993300
C	-0.89627800	2.71989800	-1.72943100
H	0.59813000	1.29303300	-2.38002200
H	1.25399300	2.87144400	-1.92398500
C	3.13623900	-0.39602900	0.22128300
H	4.34223400	-0.97609600	-1.49031000
C	1.35786600	2.26672600	2.38422000
H	2.32346100	2.98580200	0.56897600
H	0.69910200	3.55731600	0.76420200
H	-1.07780600	3.14845200	-2.72600800
H	-1.67742500	1.96856500	-1.54590300

H	-1.03545000	3.52488600	-0.99253300
C	1.93448700	-0.37053800	-0.40666900
H	3.18187100	-0.15244000	1.28910400
H	0.36610000	1.94664100	2.73754600
H	1.68073800	3.10483100	3.01985000
H	2.04952900	1.43191500	2.56944100
C	0.63226200	0.05393100	0.21392400
H	1.90637900	-0.60347300	-1.47768500
C	-0.59255900	-0.66745700	-0.36400300
H	0.68004400	-0.10314900	1.30308700
H	-0.45432100	-1.76095500	-0.29678800
H	-0.68617800	-0.43654000	-1.43910600
C	-1.88579300	-0.27822500	0.35759200
H	-1.81330600	-0.57703600	1.41787800
H	-1.98511800	0.82141500	0.36245700
C	-3.14038400	-0.89542500	-0.26293000
H	-3.04125400	-1.99520300	-0.27250800
H	-3.21005300	-0.58688800	-1.32109200
C	-4.43226500	-0.50868800	0.46200300
H	-4.36394000	-0.81962100	1.51941200
H	-4.52653600	0.59158300	0.47368600
C	-5.68996000	-1.11700900	-0.16394500
H	-5.59648700	-2.21747000	-0.17530900
H	-5.75751400	-0.80680300	-1.22182400
C	-6.98320600	-0.72811700	0.55901700
H	-6.91353600	-1.03813500	1.61556900
H	-7.07411300	0.37138600	0.56978100
C	-8.23368300	-1.34180000	-0.07609000
H	-8.18057700	-2.44133300	-0.07009500

H	-9.14776400	-1.04801800	0.45976400
H	-8.34204300	-1.02176500	-1.12378600
H	5.61818600	-0.52213000	1.27522000
C	6.88620900	-1.12072800	-0.43925600
H	6.73219700	-1.29306800	-1.51598100
H	7.58371800	-0.26858600	-0.35175600
C	7.54224000	-2.35551500	0.20470200
H	6.90221200	-3.24211400	0.09102800
H	8.51488400	-2.57189500	-0.25943300
H	7.70840400	-2.19700300	1.28042800

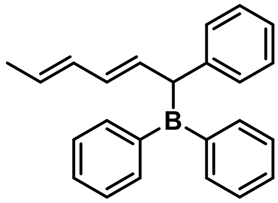

Sum of electronic and thermal Free Energies= -953.330468

B	0.06330900	0.56668100	-0.16459100
C	-0.45742400	-0.49608300	-1.24474000
C	0.32586500	-1.58381900	-0.56687000
C	1.66121600	-1.78255200	-0.72169000
H	-0.19335100	-2.17634900	0.19061000
C	2.42329200	-2.76308900	0.01245800
H	2.21238400	-1.16567700	-1.43937300
C	3.75230200	-2.95424800	-0.14368600
H	1.87723400	-3.37534700	0.73973800
C	4.56880100	-3.95401900	0.60731900
H	4.28256200	-2.33172100	-0.87347100
H	5.37845800	-3.46118600	1.17053900
H	3.95651700	-4.53042100	1.31406900
C	-0.61600100	0.59916100	1.25083600

C	-1.93049000	1.08474200	1.40325700
C	0.05457700	0.15634100	2.40912500
C	-2.54509400	1.13544700	2.65800800
H	-2.48229300	1.42904500	0.52673600
C	-0.56333300	0.18252100	3.66271400
H	1.07326400	-0.22822900	2.32269900
C	-1.86576200	0.67746800	3.79142200
H	-3.56134200	1.52294000	2.74905300
H	-0.02641800	-0.17902300	4.54154900
H	-2.34834900	0.70524600	4.76964100
C	1.24152100	1.54302400	-0.50030000
C	1.80333700	1.65443200	-1.79179200
C	1.76316800	2.39198200	0.50200900
C	2.83908700	2.54806700	-2.06627000
H	1.42254100	1.03276200	-2.60389500
C	2.80190200	3.28716500	0.23816600
H	1.34092100	2.34444700	1.50703300
C	3.34514300	3.36563800	-1.04847100
H	3.25282300	2.61176800	-3.07396500
H	3.18712100	3.92727400	1.03358500
H	4.15532200	4.06520400	-1.26032500
H	5.06295400	-4.65989200	-0.08056300
H	-0.04356700	-0.28452400	-2.23932200
C	-1.94951800	-0.65118500	-1.34126100
C	-2.63558500	0.10653100	-2.30726500
C	-2.70653400	-1.45675300	-0.47585100
C	-4.02768500	0.06546000	-2.40554100
H	-2.06227600	0.74172100	-2.98650600
C	-4.10074900	-1.50280500	-0.57333200

H	-2.21469100	-2.04729100	0.29678300
C	-4.77003900	-0.74119200	-1.53529200
H	-4.53443400	0.66427200	-3.16417300
H	-4.66613700	-2.13546400	0.11291500
H	-5.85777000	-0.77635700	-1.60812900

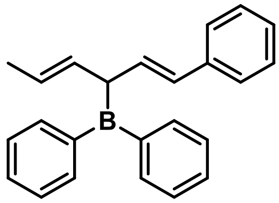

Sum of electronic and thermal Free Energies= -953.325268

B	-1.21018600	-0.11386300	-0.00297100
C	2.21613800	-1.33179700	-0.20768200
C	1.25789400	-0.55121800	-0.74377400
C	-0.16924800	-0.93914600	-0.89346700
H	1.50862800	0.46429300	-1.06573000
C	-0.82560700	-0.41850900	-2.15816400
H	-0.29851900	-2.02412400	-0.78770700
C	-1.97707700	-0.87809500	-2.68441700
H	-0.36302300	0.47137500	-2.59835200
C	-2.65603800	-0.29292000	-3.88090800
H	-2.46447000	-1.74004400	-2.21741600
H	-2.75415300	-1.03911700	-4.68580600
H	-2.10795700	0.57372300	-4.27450600
H	-3.68080900	0.02696700	-3.63118600
C	-0.98562300	1.42938300	0.19532100
C	0.11382600	1.90312700	0.94033200
C	-1.86869300	2.38728900	-0.34330900
C	0.31906600	3.27110900	1.14460000
H	0.81866700	1.18972300	1.37223400

C	-1.65606500	3.75687400	-0.16222400
H	-2.72902700	2.05223000	-0.92670800
C	-0.56224600	4.20334300	0.58741700
H	1.17370000	3.60992700	1.73276400
H	-2.34750600	4.47751900	-0.60248900
H	-0.39781100	5.27165300	0.73611700
C	-2.44216100	-0.82474400	0.65619400
C	-2.65115100	-2.22033900	0.58809600
C	-3.38014600	-0.07073200	1.39691400
C	-3.74411500	-2.82898700	1.20588700
H	-1.94497800	-2.84809800	0.04221000
C	-4.47744600	-0.67054400	2.01861200
H	-3.24021300	1.00767400	1.48597300
C	-4.66420400	-2.05328000	1.92155800
H	-3.88001300	-3.90929800	1.13439600
H	-5.18654500	-0.06172800	2.58192900
H	-5.51915000	-2.52695700	2.40668600
H	1.95103900	-2.35369600	0.08619500
C	3.61131000	-0.95240400	0.03130900
C	4.53654000	-1.94299500	0.42001500
C	4.08207100	0.37238200	-0.09798300
C	5.87767900	-1.63149100	0.65413800
H	4.19007600	-2.97249500	0.53467900
C	5.42077300	0.68326600	0.13358700
H	3.38936400	1.16985400	-0.37073700
C	6.32899100	-0.31603000	0.50926600
H	6.57219300	-2.41873600	0.95182800
H	5.75970500	1.71532800	0.02900600
H	7.37493000	-0.06791300	0.69357600

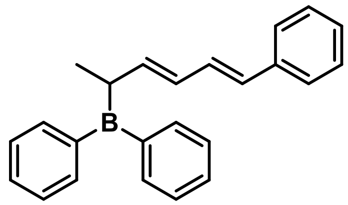

Sum of electronic and thermal Free Energies= -953.332097

B	-2.23804500	0.17084700	0.67602400
C	2.87883900	0.46637800	0.65647700
C	1.82013000	-0.30431200	1.02376200
C	0.56932500	0.23625700	1.47604500
H	1.89144000	-1.39548600	0.97461900
C	-0.49970400	-0.52408400	1.83987500
H	0.48499800	1.32745600	1.51675900
C	-1.86107800	-0.00437900	2.21363100
H	-0.41261400	-1.61282500	1.75165200
C	-2.66789300	-0.98210900	3.07101600
H	-1.76783100	0.96624700	2.72076000
H	-2.19461600	-1.13073600	4.05343500
H	-2.74928200	-1.96391400	2.58321200
H	-3.68760500	-0.60890000	3.23972900
C	-2.64605600	-1.09358400	-0.16811700
C	-3.89147700	-1.72333100	0.02820500
C	-1.79538000	-1.63031000	-1.15681600
C	-4.27522400	-2.83293300	-0.73218400
H	-4.57958700	-1.33478600	0.78192000
C	-2.16323900	-2.75245500	-1.90456700
H	-0.82336200	-1.16488200	-1.33450000
C	-3.40864000	-3.35559400	-1.69738700
H	-5.24997700	-3.29487300	-0.56539600
H	-1.47971900	-3.15364100	-2.65507800

H	-3.70172700	-4.22707200	-2.28482100
C	-2.14663300	1.57029600	-0.02814100
C	-1.86895000	2.76428900	0.67519300
C	-2.37970000	1.68570300	-1.41743700
C	-1.80774300	3.99968700	0.02955300
H	-1.69797600	2.72973700	1.75250800
C	-2.31978700	2.91710200	-2.07342800
H	-2.61190500	0.78676600	-1.99076100
C	-2.02991000	4.07911600	-1.35066000
H	-1.58959000	4.90402000	0.60001200
H	-2.50091400	2.97339800	-3.14811700
H	-1.98281000	5.04392900	-1.85825200
H	2.75992900	1.55358200	0.71882700
C	4.17592200	0.00931600	0.17580000
C	4.51353300	-1.35562700	0.03199700
C	5.15200500	0.97011200	-0.16792800
C	5.76965800	-1.73508300	-0.43428700
H	3.78557300	-2.12630600	0.28790000
C	6.41014600	0.58870400	-0.63515400
H	4.90840000	2.02944700	-0.06305100
C	6.72619400	-0.76689000	-0.77115100
H	6.00873600	-2.79462400	-0.53736500
H	7.14628300	1.35112900	-0.89394600
H	7.70838900	-1.06951100	-1.13592900

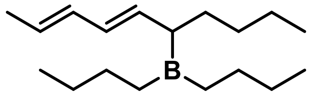

Sum of electronic and thermal Free Energies= -731.739165

B	0.54217500	0.15940400	0.40619900

C	0.90207000	-1.39110600	0.26737100
C	1.12603400	1.16684100	-0.66180900
C	-0.25088900	-1.69095200	-0.64936400
C	-0.35736900	0.63591400	1.61881200
C	0.50580500	2.55869900	-0.83665500
H	1.23549600	0.68158200	-1.64807300
H	2.17934700	1.29765300	-0.32630600
C	-1.49584300	-2.05809100	-0.25558000
H	-0.07637700	-1.51498000	-1.71768300
C	-1.78224200	1.11382900	1.26115200
H	-0.43846200	-0.15237000	2.38739700
H	0.16909500	1.48258800	2.10332000
H	-0.50648900	2.46132800	-1.26427300
H	0.37147700	3.03699300	0.14872400
C	-2.61590100	-2.24666900	-1.14924700
H	-1.68324800	-2.22360500	0.81186500
H	-1.73699500	1.91037500	0.50234300
H	-2.33296600	0.28247700	0.79158200
C	-3.85798200	-2.59569200	-0.74824700
H	-2.42932800	-2.08215200	-2.21752600
C	-5.03164900	-2.78396500	-1.65475500
H	-4.03195000	-2.75192700	0.32276800
H	-5.85508200	-2.10076700	-1.38825700
H	-4.76407600	-2.60685000	-2.70561100
C	-2.57368600	1.62085400	2.47076300
H	-2.02963700	2.46365400	2.93046900
H	-2.60956500	0.82695200	3.23626500
C	1.33273000	3.48784200	-1.73164800
H	2.34328300	3.59424600	-1.30064700

H	1.47207000	3.00936400	-2.71630100
C	0.69902600	4.86933600	-1.91312500
H	0.57707100	5.37781600	-0.94427100
H	1.31084900	5.51797100	-2.55665600
H	-0.29880200	4.78856600	-2.37125300
C	-3.99629600	2.05608600	2.10974300
H	-4.56503700	1.22040900	1.67363900
H	-4.54939300	2.41395000	2.99027200
H	-3.98411000	2.86974700	1.36826500
H	-5.44220100	-3.80381600	-1.56988300
H	0.74399100	-1.90797900	1.22849900
C	2.26798900	-1.77026200	-0.31874400
H	2.28735700	-2.84921500	-0.55379700
H	2.41851200	-1.24742700	-1.27854900
C	3.42657600	-1.43980600	0.62583500
H	3.39923700	-0.36523700	0.87850300
H	3.28336400	-1.97789800	1.57951400
C	4.80187700	-1.78606900	0.04913300
H	4.93989200	-1.24404300	-0.90185800
H	4.82787400	-2.85929700	-0.20528400
C	5.95251000	-1.45443900	1.00284300
H	6.92879400	-1.71023800	0.56653300
H	5.96555800	-0.38114400	1.24719400
H	5.85300900	-2.00783900	1.94921500

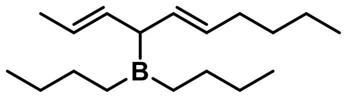

Sum of electronic and thermal Free Energies= -731.731395

B	-0.94610600	0.32034300	-0.33467500

C	2.10734200	0.02085400	-0.09875300
C	-1.71371800	-0.43237500	-1.49365000
C	1.03175800	-0.78241600	-0.02381400
C	-0.95701700	1.89978600	-0.32464200
C	-3.19958800	-0.59297600	-1.08351700
H	-1.30350300	-1.43554200	-1.69700800
H	-1.67574900	0.12967100	-2.44284300
C	-0.20916800	-0.51967900	0.80673800
H	0.99535000	-1.67011600	-0.66596700
C	-0.46743900	2.65594200	0.91620100
H	-0.39174600	2.23803200	-1.21576300
H	-1.99732200	2.19428400	-0.56172000
H	-3.26154700	-1.12260200	-0.11744100
H	-3.64748100	0.40137200	-0.91418700
C	-0.90975200	-1.76675500	1.24627900
H	0.04204500	0.10851800	1.67359100
H	-1.04734700	2.33048100	1.79776600
H	0.57720200	2.37620800	1.12691900
C	-1.35818300	-2.00660100	2.48690300
H	-1.08695400	-2.52234700	0.47046700
C	-2.10866600	-3.23659500	2.90515700
H	-1.17977400	-1.25254200	3.26321900
H	-1.59114500	-3.76836200	3.72025400
H	-2.23124800	-3.93486800	2.06491500
C	-0.55137400	4.18576400	0.80139200
H	-0.05532100	4.63906400	1.67536000
H	0.02539100	4.51082800	-0.08186700
C	-1.98303100	4.72278800	0.70455700
H	-2.57799600	4.41065000	1.57706500

H	-1.99681100	5.82152700	0.66548800
H	-2.49661700	4.35483800	-0.19544700
C	-4.03621500	-1.34861400	-2.12194000
H	-3.97141600	-0.82087200	-3.08839700
H	-3.58846500	-2.34334900	-2.28531300
C	-5.50230400	-1.50094900	-1.70906900
H	-6.08247000	-2.04756800	-2.46657900
H	-5.58984600	-2.05026100	-0.75914500
H	-5.97757300	-0.51806400	-1.56814900
H	-3.11176400	-2.98510500	3.28761500
H	2.14549000	0.91477500	0.53525900
C	3.30356000	-0.22504300	-0.96694500
H	3.12840600	-1.10911500	-1.60155300
H	3.44278100	0.63280100	-1.65034900
C	4.60051100	-0.41330800	-0.15969100
H	4.75212600	0.46198600	0.49530900
H	4.48417500	-1.28221800	0.50996200
C	5.83510400	-0.60755600	-1.04476700
H	5.67444600	-1.47786100	-1.70324000
H	5.94151700	0.26467800	-1.71174200
C	7.12162100	-0.80152100	-0.23838100
H	7.04984200	-1.68554700	0.41342500
H	7.99352400	-0.93888100	-0.89391000
H	7.31968100	0.06931400	0.40503200

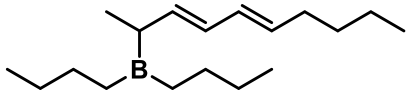

Sum of electronic and thermal Free Energies= -731.738749

B	-2.11412000	-0.06957800	0.09229100

C	3.22001700	-0.01949700	-0.32078600
C	-2.83721400	-1.26278500	-0.65427100
C	2.11770800	-0.72852800	0.00777500
C	-2.07273000	1.33980300	-0.62328000
C	-4.35607400	-1.20833300	-0.35418300
H	-2.46087200	-2.25197900	-0.34376200
H	-2.70213500	-1.19666600	-1.74816000
C	0.93669700	-0.16359100	0.62031900
H	2.09916900	-1.80529600	-0.20105400
C	-1.66514300	2.58500300	0.17176800
H	-1.41450200	1.23178300	-1.50860800
H	-3.07171300	1.50315900	-1.07386900
H	-4.52480000	-1.25728000	0.73512900
H	-4.76418100	-0.23616900	-0.68024200
C	-0.16796300	-0.87514600	0.95481000
H	0.95608900	0.91310900	0.82493800
H	-2.32314100	2.69445500	1.05169600
H	-0.64756600	2.45603600	0.57772200
C	-1.44447000	-0.30855800	1.51768400
H	-0.17664700	-1.94945700	0.73445700
C	-2.14818300	-1.27229900	2.48198200
H	-1.22652500	0.64559200	2.02068600
H	-1.52174400	-1.48742900	3.36150600
H	-2.37554600	-2.23146400	1.99345500
H	-3.09741800	-0.84845900	2.83898700
C	-5.14782900	-2.33399000	-1.02964300
H	-4.98078800	-2.28481800	-2.11885800
H	-4.74017500	-3.30468900	-0.70072700
C	-6.64765000	-2.27235300	-0.72995000

H	-7.08138600	-1.32174600	-1.07642000
H	-7.19388400	-3.08894900	-1.22392700
H	-6.83798300	-2.34843600	0.35163500
C	-1.70621000	3.87900800	-0.64738100
H	-2.72438400	4.01827700	-1.04974000
H	-1.04643400	3.76806800	-1.52510800
C	-1.29386500	5.11470200	0.15646700
H	-1.33131400	6.02933500	-0.45284300
H	-1.95758500	5.26198500	1.02243200
H	-0.26761100	5.01049900	0.54126400
H	3.23175300	1.05624000	-0.10547400
C	4.45848900	-0.58859800	-0.93748800
H	4.30483700	-1.65482200	-1.17068800
H	4.66129700	-0.07940100	-1.89770500
C	5.70087800	-0.42881000	-0.04026600
H	5.52576700	-0.95454300	0.91349200
H	5.83110700	0.63701800	0.21456400
C	6.98171000	-0.95787600	-0.69226400
H	6.84211900	-2.02042100	-0.95354800
H	7.14648600	-0.42776700	-1.64552600
C	8.21296200	-0.80457300	0.20412300
H	8.08353500	-1.35171900	1.15035900
H	9.11942900	-1.19015700	-0.28418200
H	8.39056400	0.25252700	0.45416700

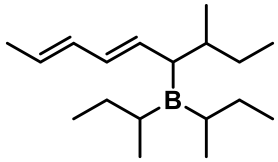

Sum of electronic and thermal Free Energies= -731.733142

B	1.00181300	-0.36802200	-0.07359200
C	0.19928100	0.97557200	-0.42606300
C	1.19834600	-0.72660500	1.46162500
C	-1.14306100	0.90044000	0.22783000
C	1.40919200	-1.28318000	-1.30298800
C	0.30837900	-1.95935900	1.78271300
H	0.79786400	0.10751000	2.06725200
C	-2.32253300	0.70465200	-0.40413200
H	-1.15416300	0.97278700	1.32270000
C	0.09972600	-1.98929400	-1.77497000
H	1.68110200	-0.58943800	-2.12309800
H	-0.68826100	-1.81798500	1.33221100
H	0.73779100	-2.85100300	1.29725300
C	-3.59790600	0.57725200	0.26956700
H	-2.33190300	0.63350100	-1.49872000
H	-0.14990700	-2.78681100	-1.05621800
H	-0.73983900	-1.27787100	-1.74081300
C	-4.78054400	0.37929400	-0.35153700
H	-3.58202800	0.64655400	1.36464500
C	-6.10079100	0.24415600	0.33943700
H	-4.78750100	0.30916100	-1.44567700
H	-6.57539600	-0.72501900	0.11250300
H	-5.99531400	0.32684500	1.43025100
C	0.13516000	-2.21958800	3.28279900
H	-0.53033800	-3.07674000	3.46162500
H	1.09497800	-2.43704300	3.77224500
H	-0.30390100	-1.34201400	3.78157500
C	2.65728300	-0.93911700	1.91351000
H	3.09251200	-1.84406900	1.46990000

H	3.29584800	-0.08973200	1.62940600
H	2.72309000	-1.03886400	3.00722200
C	0.18782000	-2.57679400	-3.18756200
H	-0.76252900	-3.04885200	-3.47723000
H	0.41156500	-1.78881900	-3.92264400
H	0.97364700	-3.34112600	-3.26597600
C	2.55738500	-2.28834500	-1.13225300
H	2.32145800	-3.04214200	-0.36612100
H	2.76707600	-2.82746200	-2.06860600
H	3.48849800	-1.78860000	-0.83083500
H	-6.81091100	1.01797700	0.00319400
H	0.05868000	1.05604400	-1.52000100
C	1.02630500	2.20804400	0.04238600
H	1.10010800	2.15814000	1.14429200
C	2.45411900	2.12609400	-0.52631900
H	2.40097600	2.17575300	-1.62787700
H	2.87380200	1.12996600	-0.29496200
C	3.41435200	3.19311500	0.00255000
H	4.43105900	3.03874700	-0.38581300
H	3.10013500	4.20524000	-0.28853100
H	3.46659500	3.16229500	1.10167800
C	0.32521600	3.51715500	-0.33420900
H	0.26501500	3.61820900	-1.42989000
H	-0.70084100	3.53840200	0.05959600
H	0.85559000	4.39560600	0.05909900

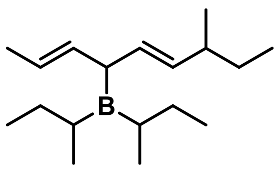

Sum of electronic and thermal Free Energies= -731.730578

B	1.12025400	0.36391600	0.01200600
C	-2.11981600	-0.34035000	-0.30932700
C	1.56574700	0.27807100	1.52820300
C	-0.96506900	-0.88171000	0.10701000
C	1.45850800	1.64192900	-0.85874200
C	3.11640500	0.20835700	1.56257000
C	0.33374500	-0.85825800	-0.65810500
H	-0.92414400	-1.32149200	1.11078100
C	0.15351200	2.35679300	-1.29426100
H	2.03392400	2.36313000	-0.24951300
H	3.45944800	-0.56950200	0.85957900
H	3.52736100	1.16102300	1.18842400
C	1.16138800	-2.10415700	-0.51191500
H	0.13628400	-0.66329000	-1.72377600
H	-0.39952900	1.70229900	-1.98798400
H	-0.50193700	2.48671300	-0.41772800
C	1.83920600	-2.70242200	-1.50300800
H	1.24264200	-2.51391600	0.50245000
C	2.71482600	-3.90868100	-1.33734400
H	1.76097800	-2.28810000	-2.51531900
H	2.37651300	-4.74347300	-1.97256000
H	2.72649700	-4.25474500	-0.29406400
H	3.75382200	-3.69338200	-1.63704700
C	0.38365400	3.72314000	-1.94819600
H	0.97476900	3.64015900	-2.87119100
H	-0.57117300	4.20252300	-2.20934700
H	0.92292100	4.39734400	-1.26533000
C	3.69338600	-0.09467700	2.94954800

H	3.30684200	-1.05147500	3.33241300
H	4.79046600	-0.16438900	2.91529900
H	3.43418000	0.68611300	3.67848900
H	1.19142700	-0.65061900	1.99506200
C	1.02227500	1.46940300	2.34210300
H	1.32463100	1.41574500	3.39867500
H	1.38945300	2.42567000	1.93883800
H	-0.07708400	1.49854400	2.31645600
C	2.32918700	1.25038100	-2.07202100
H	3.24729400	0.73194400	-1.75860000
H	1.78334000	0.57545500	-2.74868800
H	2.63424100	2.13214400	-2.65489400
H	-2.16586700	0.11273300	-1.30821800
C	-3.38873600	-0.27733000	0.49506000
H	-3.20800700	-0.78160900	1.46123200
C	-4.52597900	-1.03062800	-0.23037500
H	-4.16540400	-2.04248500	-0.47541300
H	-4.72060800	-0.53079200	-1.19523800
C	-5.82524800	-1.13594400	0.57281400
H	-6.56970900	-1.74316400	0.03837900
H	-5.64618600	-1.61002100	1.55013500
H	-6.27554800	-0.15051600	0.75755300
C	-3.75613100	1.19191700	0.77518200
H	-2.93587500	1.70443600	1.29685700
H	-3.94403400	1.72861300	-0.16810100
H	-4.65836700	1.27379700	1.39718100

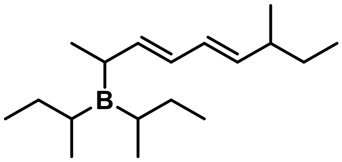

Sum of electronic and thermal Free Energies= -731.735173

B	-2.10675500	0.09581300	0.01937900
C	3.43984500	0.31732800	0.12414700
C	-2.67056300	-1.29024400	-0.50214200
C	2.30676200	-0.37061300	0.38077900
C	-2.26029900	1.40046800	-0.87841300
C	-4.20234400	-1.21764000	-0.22785300
H	-2.27972100	-2.12068300	0.11310000
C	1.06424600	0.23129800	0.81157600
H	2.30875400	-1.46007800	0.25243900
C	-2.60743300	2.67957300	-0.09659200
H	-3.08603400	1.21891800	-1.59300300
H	-4.38763000	-0.77713000	0.76666300
H	-4.65853300	-0.52389600	-0.95335200
C	-0.07452000	-0.45653100	1.06357600
H	1.06211400	1.32042200	0.93795900
H	-2.67612900	3.52792800	-0.80019700
H	-1.77947600	2.92545300	0.58996400
C	-1.39575100	0.14924200	1.44747200
H	-0.06190200	-1.54573500	0.93503400
C	-2.08674300	-0.63212500	2.57920400
H	-1.22589600	1.18912200	1.76838100
H	-1.46941100	-0.63468300	3.49048100
H	-2.26399300	-1.67944200	2.29416600
H	-3.05977500	-0.18637100	2.83114100
C	-4.90397400	-2.57844000	-0.29363900

H	-5.98168700	-2.47926400	-0.09761900
H	-4.78766000	-3.04687000	-1.28117000
H	-4.48640600	-3.26772600	0.45558600
C	-3.91353400	2.56355100	0.69284400
H	-3.85440700	1.76077500	1.44430500
H	-4.15615900	3.49590400	1.22274000
H	-4.75599600	2.32564500	0.02552800
C	-0.98333100	1.62069900	-1.72663900
H	-1.13717700	2.44324900	-2.44341800
H	-0.12992300	1.88614600	-1.08749700
H	-0.70079700	0.72482300	-2.29659400
C	-2.37922400	-1.61028500	-1.97701600
H	-2.75264200	-0.81374600	-2.63880200
H	-1.29883900	-1.71137700	-2.15610100
H	-2.85238400	-2.55198400	-2.29478200
H	3.43285700	1.40830600	0.24909100
C	4.73849300	-0.28387600	-0.32763300
H	4.59894700	-1.37557500	-0.42102500
C	5.83710800	-0.03523500	0.73309200
H	5.45920200	-0.38415600	1.70724200
H	5.99062400	1.05316800	0.83298600
C	7.17031000	-0.72446500	0.42980300
H	7.88434600	-0.57456500	1.25209500
H	7.03220800	-1.80878900	0.29976800
H	7.63604700	-0.33456600	-0.48602300
C	5.13359000	0.27773200	-1.70649800
H	4.34221900	0.09062700	-2.44549200
H	5.29332600	1.36583900	-1.64837900
H	6.05988200	-0.18137900	-2.07851300

References

[1] X. Wang, N. Hadjichristidis, *Angew. Chem. Int. Ed.* **2021**, *60*, 8431-8434.

[2] M. J. Frisch, G. W. Trucks, H. B. Schlegel, G. E. Scuseria, M. A. Robb, J. R. Cheeseman, G. Scalmani, V. Barone, G. A. Petersson, H. Nakatsuji, X. Li, M. Caricato, A. V. Marenich, J. Bloino, B. G. Janesko, R. Gomperts, B. Mennucci, H. P. Hratchian, J. V. Ortiz, A. F. Izmaylov, J. L. Sonnenberg, Williams, F. Ding, F. Lipparini, F. Egidi, J. Goings, B. Peng, A. Petrone, T. Henderson, D. Ranasinghe, V. G. Zakrzewski, J. Gao, N. Rega, G. Zheng, W. Liang, M. Hada, M. Ehara, K. Toyota, R. Fukuda, J. Hasegawa, M. Ishida, T. Nakajima, Y. Honda, O. Kitao, H. Nakai, T. Vreven, K. Throssell, J. A. Montgomery Jr., J. E. Peralta, F. Ogliaro, M. J. Bearpark, J. J. Heyd, E. N. Brothers, K. N. Kudin, V. N. Staroverov, T. A. Keith, R. Kobayashi, J. Normand, K. Raghavachari, A. P. Rendell, J. C. Burant, S. S. Iyengar, J. Tomasi, M. Cossi, J. M. Millam, M. Klene, C. Adamo, R. Cammi, J. W. Ochterski, R. L. Martin, K. Morokuma, O. Farkas, J. B. Foresman, D. J. Fox, Wallingford, CT, **2016**.

[3] S. Grimme, J. Antony, S. Ehrlich, H. Krieg, *J. Chem. Phys.* **2010**, *132, 154104*.

[4] (a) A. D. Becke, *Phys. Rev. A* **1988**, *38*, 3098-3100; (b) J. P. Perdew, *Phys. Rev. B* **1986**, *33*, 8822-8824; (c) V. N. Staroverov, G. E. Scuseria, J. M. Tao, J. P. Perdew, *J. Chem. Phys.* **2003**, *119*, 12129-12137; (d) F. Furche, J. P. Perdew, *J. Chem. Phys.* **2006**, *124, 044103*.

[5] (a) F. Weigend, R. Ahlrichs, *Phys. Chem. Chem. Phys.* **2005**, *7*, 3297-3305; (b) F. Weigend, *Phys. Chem. Chem. Phys.* **2006**, *8*, 1057-1065.

[6] M. Cossi, N. Rega, G. Scalmani, V. Barone, *J. Comput. Chem.* **2003**, *24*, 669-681.
